# The association of DNA damage response and nucleotide level modulation with the antibacterial mechanism of the anti-folate drug Trimethoprim

**DOI:** 10.1186/1471-2164-12-583

**Published:** 2011-11-28

**Authors:** Dipen P Sangurdekar, Zhigang Zhang, Arkady B Khodursky

**Affiliations:** 1 Lewis-Sigler Institute for Integrative Genomics, Princeton University, 132 Carl C. Icahn Laboratory, Princeton University, Washington Road, Princeton NJ 08540, USA; 2 Department of Biochemistry, Molecular Biology and Biophysics, University of Minnesota, 1479 Gortner Ave, Saint Paul, MN 55108, USA; 3 Biotechnology Institute, University of Minnesota, 1479 Gortner Ave, Saint Paul, MN 55108, USA; 4 Metabolix Inc., 21 Erie Street, Cambridge, MA 02139, USA

## Abstract

**Background:**

Trimethoprim is a widely prescribed antibiotic for a variety of bacterial infections. It belongs to a class of anti-metabolites - antifolates - which includes drugs used against malarial parasites and in cancer therapy. However, spread of bacterial resistance to the drug has severely hampered its clinical use and has necessitated further investigations into its mechanism of action and treatment regimen. Trimethoprim selectively starves bacterial cells for tetrahydrofolate, a vital cofactor necessary for the synthesis of several metabolites. The outcome (bacteriostatic or bactericidal) of such starvation, however, depends on the availability of folate-dependent metabolites in the growth medium. To characterize this dependency, we investigated in detail the regulatory and structural components of *Escherichia coli *cellular response to trimethoprim in controlled growth and supplementation conditions.

**Results:**

We surveyed transcriptional responses to trimethoprim treatment during bacteriostatic and bactericidal conditions and analyzed associated gene sets/pathways. Concurrent starvation of all folate dependent metabolites caused growth arrest, and this was accompanied by induction of general stress and stringent responses. Three gene sets were significantly associated with the bactericidal effect of TMP in different media including LB: genes of the SOS regulon, genes of the pyrimidine nucleotide biosynthetic pathway and members of the multiple antibiotic resistance (mar) regulon controlled by the MarR repressor. However, the SOS response was identified as the only universal transcriptional signature associated with the loss of viability by direct thymine starvation or by folate stress. We also used genome-wide gene knock-out screen to uncover means of sensitization of bacteria to the drug. We observed that among a number of candidate genes and pathways, the effect of knock-outs in the deoxyribose nucleotide salvage pathway, encoded by the *deoCABD *operon and under the control of the DeoR repressor, was most informative.

**Conclusion:**

Transcriptional induction of DNA damage response is an essential feature of the bactericidal effect of trimethoprim. Either the observation of the transcriptional response or DNA damage itself, or both, is made possible by thymine starvation when other folate-dependent metabolites are not limited. The effect of DNA damage by the drug takes place prior to its bactericidal effect, at the beginning of the lag stage of the treatment. Mutations in the deoxyribose nucleotide salvage pathway can affect duration of the lag as well as the rate of killing. This information can be used to postulate certain mechanistic differences between direct thymine starvation in thymidylate synthase deficient mutants and thymine starvation by anti-folate inhibitors.

## Background

2,4,-diamino-5-(3', 4', 5'-trimethoxybenzyl)-pyrimidine (Trimethoprim, TMP) is a folate analog that inhibits the reduction of dihydrofolate to tetrahydrofolate (THF) by competitively binding the active site of the enzyme dihydrofolate reductase (DHFR) [1,2]. The drug is highly specific against bacterial and malarial enzymes [3], has a wide antibacterial spectrum and, despite emerging resistance [4-7], has been extensively used against urinary and respiratory tracts infections and against enteric pathogens and methicillin resistant *S. aureus *[8].

**Figure 1:**
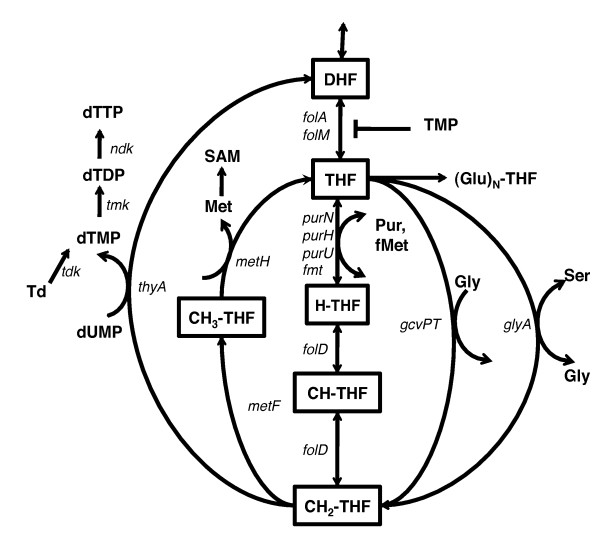
**Overview of tetrahydrofolate metabolism**. DHF: dihydrofolate; THF: tetahydrofolate (glu)_1_; H-THF: formyl-tetrahydrofolate; CH-THF: 5,10-methenyltetrahydrofolate; CH_2_-THF: 5,10-methylenetetrahydrofolate; CH_3_-THF: 5-methyltetrahydrofolate; Met: methionine; Ser: serine; Gly: glycine; SAM: S-adenosylmethionine; Td: thymidine; Pur: Purines; fMet: N-methionyl-tRNA^fmet^.

Trimethoprim treatment reduces the pool of 5,6,7,8-tetrahydrofolate (THF) by inhibiting DHFR, encoded by the *folA *and *folM *genes in *E. coli *(Figure 1). THF is methylated during the synthesis of glycine from serine and gets converted to 5,10-methylenetetrahydrofolate (CH_2_-THF), which is a major one-carbon donor in the cell for the synthesis of methionine and S-adenosylmethionine (SAM), purines, N-formylmethionine-tRNA (fMet-tRNA) and thymidylate (dTTP) [9,10]. Thus TMP treatment leads to the loss of THF pools to dihydrofolate because of thymidylate synthesis. This causes concomitant starvation of glycine, methionine, purines, dTTP (folate dependent metabolites) and fMet-tRNA, with some metabolites being depleted faster than others [11,12], and results in nearly complete cessation of DNA, RNA and protein synthesis [11,13,14]. Other pathways affected due to depletion of SAM, a major methyl group donor, include synthesis of cofactors, fatty acids and polyamines. Starvation of cells for purines and amino acids is usually bacteriostatic; starvation of dTTP alone leads to loss of viability and is known as "thymineless death" [15-17]. Therefore, cellular outcome of TMP treatment depends on the balance of folate dependent metabolites in the cell and on the media supplementation conditions; cell death depends upon the right combination of supplementation regimes resulting, in part, in thymine starvation [15,16]. Thus in controlled environment in the laboratory, the outcome of TMP treatment can in principle be correlated with the composition of the growth medium. However, despite a general understanding of the drug mechanism, the following key aspects of the antibacterial activity of TMP remains unknown: i) what is the molecular nature of folate stress induced by the drug?; ii) what is the relationship between the folate stress and media composition?; iii) what are the similarities and differences between bacateriostatic and bactericidal signatures of the TMP effects.

To answer these and related questions, we treated *Escherichia coli *with TMP in a variety of media and supplementation conditions and tracked global transcriptional responses using microarrays. We also applied a genome-wide genetic screen to identify gene knock-out mutants with decreased or increased susceptibility to TMP in rich media. We observed that the drug treatment, either in minimal medium supplemented with requisite amino acids and adenine or in complex medium (LB), resulted in the loss of viability and this loss was correlated with the induction of DNA damage repair genes. Under limited supplementation conditions (only amino acids or purines) in minimal media, the primary signature was that of stress response, but when both were added in absence of thymine, the stress response signature was not observed. Addition of individual supplements (amino acids or purine) also led to the repression of the respective biosynthetic genes, even in the presence of TMP. We also identified a signature of gene set activity, including induction of DNA damage response, pyrimidine nucleotide biosynthesis and antibiotic stress responsive MarR targets to be most associated with cell lethality in minimal media. However, only the SOS response was associated with the bactericidal outcome across all tested nutrient conditions, including thymine starvation in a thymine auxotroph.

Using a genome-wide collection of knock-out mutants to screen for genetic, rather than environmental, factors that can affect TMP susceptibility, we found that the deoxyribose nucleotide salvage pathway has the capacity to modulate and mediate the effect of the drug. The *deoA *and *deoC *gene knock-out mutants were found to be resistant to TMP in complex medium, whereas the loss of their transcriptional repressor *deoR *rendered bacteria more sensitive to the drug. However, this relationship was reversed in minimal medium with supplements. This reversal was also observed at the transcriptional level wherein the *deoCA *genes were down-regulated during TMP treatment in LB medium and up-regulated in M9 minimal medium. Using this data and other available information we propose that the nucleotide salvage pathway encoded by *deoCABD *genes is a critical modulator of physiological outcomes of the TMP treatment in *E. coli*.

## Methods

### Growth conditions

*Escherichia coli *MG1655 *F^- ^lambda^- ^ilvG^- ^rfb-50 rph^- ^*was used as a wild-type strain in all experiments. Trimethoprim (Fluka (Sigma-Aldrich), St. Louis, MO) stock solution was prepared by dissolving the drug in distilled water with 1% v/v glacial acetic acid (for Keio screen) or by dissolving in 50% v/v ethanol-chloroform mixture (for transcriptional profiling) and stored in small aliquotes at -20°C. Complex medium for cell growth was LB (Luria Bertani broth) and minimal medium was M9 minimal medium with 0.4% glucose [18]. In LBTMP experiments, cells were treated with 50 μg/ml of TMP, while for M9 experiments the concentration used was 25 μg/ml, corresponding to the MIC_50 _determined in the respective media after 18 hrs of treatment. Amino acids for supplementation (methionine and glycine) were purchased from Fluka (Sigma-Aldrich, St. Louis, MO) and stock solutions were prepared in water. Standard cultures were grown in flasks at 37°C under aeration in a rotary shaker either in M9 minimal medium containing 0.4% glucose or in LB liquid medium. For M9 with folate dependent supplementation experiments, the following chemicals were added with a final concentration of 50 μg/ml: adenine, amino acid (methionine and glycine) mixture, and thymine.

### Microarray experiments

Overnight cultures were resuspended in fresh medium and TMP was added when the optical density (O.D. 600 nm) of the culture reached 0.3-0.4. Time-point samples were taken every 15-30 minutes from 15 minutes to 2 hours post treatment. The samples were spun down and snap-frozen in liquid nitrogen before storing at -80°C until required. Total RNA samples were purified using the Qiagen RNeasy kit (Chatsworth, CA) according to the manufacturer's protocol. To identify genome-wide gene expression profiles, relative mRNA levels were determined by parallel two color hybridization at single-gene resolution to whole-genome *E. coli *K-12 MG1655 spotted DNA microarrays, designed, printed and probed as described [19], and containing discrete sequence elements corresponding to 98 and 3/4% of all annotated open reading frames (ORFs). Fluorescence DNA probes were synthesized from 10~15 μg of total RNA with random hexamers and Cy-5 dUTP or Cy-3 dUTP dyes (Amersham) and hybridized to microarrays for 6 hours at 65°C followed by a washing step as described previously [19]. All microarray experiments were designed as time courses, using the 0 min time point sample as a common reference. The hybridized arrays were then scanned and the raw fluorescence intensity data were normalized in R (http://cran.r-project.org/) using the Limma (Bioconductor) package [20] to remove spatial and array based biases. The data was converted to log-ratios of normalized intensity at each time point to time point 0. The microarray data was uploaded to NCBI GEO database and is available for download through accession number GSE32562.

### Gene Set Analysis

To analyze gene expression data in the context of known gene sets and pathways, we obtained gene functional classification information from databases (GenProtEC [21], RegulonDB [22], EcoCyc [23]) to assemble a total of 480 gene sets (Additional File 1). These comprise of sets of genes whose products are involved in the same pathway and sets of genes that share common regulators or other properties. Normalized gene expression data was analyzed for gene set activity using a method modified from Tian et al [24]. We then modeled the normalized set scores across the entire time series to analyze the effect of individual supplements on gene set activity and to infer set activity associated with the lethal phenotype. The raw gene set score for *k^th ^*gene set was calculated as:

$$S_{k}=\overset{N_{k}}{\underset{i=1}{\sum }}w_{i}T_{i}$$

where *w_i _*corresponds to the contribution of gene i within a set to the principal eigenvector of the set across a compendium of experimental conditions [25] and *N_k _*is the number of genes in gene set *k*. The weights allow us to consider sets that only have a subset of actively transcribed genes or sets with genes showing opposite but coordinated trends [26,27]. The score *S *was normalized under two sets of hypothesis *Q_1 _*and *Q_2 _*[24], corresponding to testing against random genes and random phenotypes, to yield two normalized gene set scores for each gene set. This was done by randomly sampling genes (in *Q_1_*) or conditions (in *Q_2_*) and then calculating the bootstrapped scores under these permutations. These null scores are normalized to mean 0 and standard deviation 1 to account for gene set size dependence of the distributions. The observed gene set scores *S_k _*were also normalized using the parameters of the null distributions. P-values calculated for both *Q_1 _*and *Q_2 _*scores were converted to posterior probability (PP) of significance, which is mathematically equivalent to the local False Discovery Rate (FDR) value of the score [28], and PP values from both hypotheses were multiplied, under assumption of independence, to yield the final PP for the gene set. PP values of greater than 0.90 (estimated FDR ~ 5%) were considered to be highly significant. The normalized scores in the two hypotheses for gene set were averaged to yield a enrichment score (ES), which allows us to quantitatively compare two or more gene sets within a condition or a gene set across conditions, and its sign indicates the direction of change (up- or down-regulation) within the set. To infer effects of individual supplementation, the set scores (ES) for the entire time series of M9 treatments were modeled as:

$$ES_{i}=\beta_{i}^{AA}AA_{i}+\beta_{i}^{Ad}Ad_{i}+\beta_{i}^{Thy}Thy_{i}+\varepsilon_{i}$$

where ES_i _is the vector of set scores of set *i*; AA_i_, Ad_i _and Thy_i _are factors with level 1 (if the respective supplements are added) or 0. M9TMP condition has all three factors at level 0 since none of the supplements were added. A positive coefficient for any factor indicates that the addition of the supplement is associated with increase in set score relative to its baseline level.

For inferring associations with cell lethality, set scores from both LB and M9 experiments were considered. To account for the influence of latent variables affecting the set scores, surrogate variable analysis (SVA, [29]) was used to model the hidden variables as a covariate:

$$ES_{i}=\beta_{i}^{SV}SV_{i}+\beta_{i}^{Phe}Phe_{i}+\varepsilon_{i}$$

where ES_i _is the vector of set scores for set *i*, SV*_i _*are surrogate variables and Phe*_i _*represents a vector of phenotypes associated with the set (factor with two levels: "cidal" or "static"). Parametric p-values associated with the estimated coefficients $\beta_{i}^{Phe}$ were obtained by ANOVA, and False Discovery Rates (q-value [30]) was calculated to infer significant gene sets. A q-value of 5% indicates all sets with the same or lower q-value are called significant at 5% False Discovery Rate. All calculations were done in R. Non-parametric classification based on Receiver-Operator Characteristics (ROC) curves were done by using the R package "caTools"[31]. AUC values of 0.85 and higher were considered significant, and it was used as the second threshold to filter significant gene sets. The entire data and the R code to reproduce the analysis can be found in Additional File 2.

### Genome-wide genetic screen

The Keio collection was a kind gift of Prof. Hirotada Mori and consists of knock-out mutants of 3985 genes of *E. coli *strain BW25113 [32]. The knock-out mutants contain Kan^R ^cassettes inserted in genes using the method of Datsenko and Wanner [33]. For mutant screening, a copy of the mutant plates was made by replica inoculating the frozen stocks into 96-well plates containing 150 *μ*l of LB medium and incubating them at 30°C for 12-18 hours with intermittent shaking. For growth and inhibition experiments, the optical density in the wells of the 96-well plate was measured using Victor3 plate reader (Perkin-Elmer). The actual O.D. reading in the plate reader depends on the medium and the volume of liquid in the wells and is correlated with actual culture density measured by conventional light scattering techniques (r^2 ^> 0.99) for O.D. 0.1-1.0. Since each plate was inoculated as a set, there was plate-to-plate variation in O.D. that was accounted for by normalizing across plates. Resistant and sensitive mutants were identified by normalized O.D. readings. Mutants in Keio collection are in BW25113 background. For individual mutant analysis, the Kan*^R ^*insertion alleles were transferred into wild-type MG1655 background using P1-transduction [34]. An *E. coli thyA715 *mutant MG1655 was obtained from CGSC. Different *deo *mutations were similarly introduced into the *thyA*^- ^parental strain. *thyA*^-^*deo*^- ^double mutants had different thymine/thymidine growth requirements; *thyA*^- ^20 *μ*g/ml thymine, *thyA*^-^*deoA*^- ^20 *μ*g/ml thymidine, *thyA*^-^*deoC*^- ^2 *μ*g/ml thymine, *thyA*^-^*deoR*^- ^50 *μ*g/ml thymine [35,36] For viability experiments and RNA sampling, the cultures were handled as follows. Cells were grown to stationary phase in LB or M9 minimal media (with glucose). They were then inoculated in fresh medium and grown till O.D. 0.4-0.6 before treatment. For TMP treatment in LB, TMP was added to appropriate final concentrations. For TMP treatment in minimal medium, growing cultures were diluted in pre-warmed fresh minimal medium containing TMP and methionine, glycine, adenine supplements. For viability counts, samples were diluted in 0.9% NaCl, spread or spotted on LB plates and incubated 12-16 hours before colony counts were taken.

## Results and Discussion

### Folate supplementation regimes determine bacterial viability phenotypes

The effect of antibiotic trimethoprim (TMP) on bacteria depends on the treatment conditions. To characterize the cellular responses associated with these effects, we treated *E. coli *with the antibiotic in different growth and supplementation conditions: complex medium (LBTMP), minimal medium M9 (M9TMP), and minimal medium supplemented with folate dependent metabolites, amino acids methionine and glycine (M9TMPAA), adenine (M9TMPAd), amino acids and adenine (M9TMPAdAA), and M9 supplemented with the amino acids, adenine and thymine (M9TMPThyAdAA) (Table 1). These conditions were chosen based on their causing different outcomes in TMP treatment: only in minimal medium the treatment results in growth arrest, supplementation with both AA and Ad leads to cell killing, and addition of thymine rescues cells from the effects of the drug [15]. We followed the effects of the drug treatment by monitoring colony formation, as a proxy to cell viability, and genome-wide transcriptional responses, as a proxy to physiological changes elicited by the drug. As expected, cell viability in minimal medium depended on supplementation of the folate-derived metabolites. TMP treatment in M9 resulted in cessation of growth without a loss of viability, whereas M9TMPAdAA condition resulted in killing (Table 1) after a lag period, along with increase in cell mass due to filamentation (data not shown). Thymine supplementation prevented viability loss in the bacterial culture, confirming that the primary mechanism of TMP induced cell death is due to thymine starvation [15]. Incomplete supplementation either by the amino acids or by the purine resulted in bacteriostasis. LBTMP condition resulted in loss of viability after a lag period of approximately 120 min, as well as in filamentation.

**Table 1:** Treatment regimens used for gene expression analysis

Condition	Base medium	Supplementation	Viability loss
LBTMP	Luria-Bertani broth	-	+ (90% loss in 3 hours)
M9TMP	Minimal M9	None	-
M9TMPAdAA	Minimal M9	Met, Gly, Ad	+ (>70% loss in 6 hours)
M9TMPAA	Minimal M9	Met, Gly	-
M9TMPAd	Minimal M9	Ad	-
M9TMPThyAdAA	Minimal M9	Met, Gly, Ad, Thy	-

A list of treatment regimens used. Viability loss indicates the relative magnitude of cell lethality (+: maximum, -: no loss). TMP: Trimethoprim (50 or 25 μg/ml), Met: methionine, Gly: glycine, Ad: adenine (each at 50 μg/ml final concentration)

### Effects of individual supplementation regimes

Given the diverse nature of phenotypes generated by the combination of TMP and media conditions, and the complex nature of folate metabolism being perturbed, it is essential to identify key molecular events during each treatment that are associated with the observed phenotype. We first followed transcriptional responses to TMP treatment in these conditions as a time series using cDNA microarrays and obtained gene expression profiles as ratios of transcript abundances after the treatment (10 - 120 minutes) normalized to the before-treatment levels (0 minutes). To develop a biological interpretation of these responses, we examined transcriptional activity of sets of genes [24]. Briefly, for each time point in each condition, the activity score (*S_k_*) for each gene set was calculated as the weighted average of all the genes within the gene set (Methods). The score was then normalized using background null distributions [24] to get the enrichment scores (ES) and the p-values for these scores were then converted to a posterior probability (PP, [28]) to adjust for multiple testing. The PP value (1 for strong enrichment, 0 for no enrichment), along with the enrichment score ES, allowed for quantitative comparison of different gene sets in different conditions. PP values of greater than 0.90 were considered to be highly significant for gene set activity, corresponding to false discovery rates of less than 5% for the conditions.

The effect of TMP in different supplementation conditions can be viewed and analyzed as an interaction between the effects of the drug effect and the condition. Traditionally, the effect of a drug would be ascertained in cultures adapted over multiple generations to their growth environments. While such approach may seem physiologically most sound, it does not allow determining the media specific effects in any straightforward way, in part because of a prolonged adaptation and resulting steady state are accompanied by macroscopic growth and metabolic differences which can mask nutrient-specific regulation. This has not been seen as a serious problem since for most actively studied antibacterials, the media composition either does not have an effect on the outcome of the treatment or its effects are not known. This however would be a serious problem for TMP treatments where media composition effects have been well documented. Therefore we designed our experiment in a way that yielded physiologically meaningful, and consistent with historical data, media-dependent outcomes of the treatments and also allowed detecting direct regulatory effects of the supplementations. According to such design, the drug was added to the cell cultures along with the supplements and transcriptional responses were recorded immediately afterwards. Thus the ensuing regulatory and physiological changes in the cells will be, by definition, a result of the combinations, or interactions, of the effects of the drug and supplements. Modeling these transcriptional responses as estimated additive effects of each supplement could help us explain these interactions as simple cause and effect representation for each individual factor-gene set pair.

**Figure 2:**
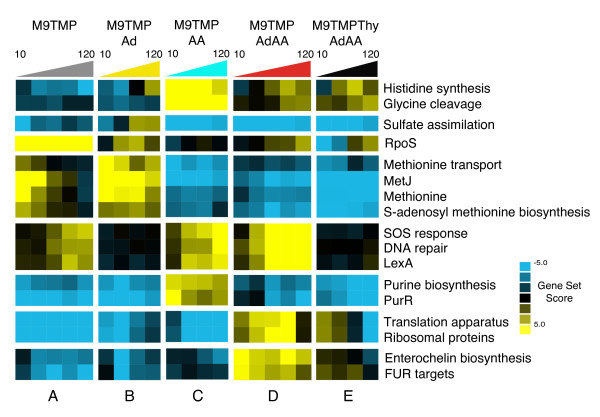
**Expression profiles of top gene sets associated with individual supplementation of folate-dependent metabolites**. Heatmap of gene set scores for top gene sets associated with individual factors. Blue and yellow color refers to down-regulation and up-regulation of gene sets respectively. Colored triangles above the heatmap indicate time progression from 10 min to 120 min after the beginning of drug treatment. The figure is split into vertical panels A-E according to the condition and into horizontal strips according to the gene set.

To analyze the effects of individual supplements (AA: met, gly, Ad: adenine and Thy: thymine) on the TMP-induced folate stress, we employed a linear model using set scores across all time points for each condition and with individual supplements as factors, to identify the gene sets with the highest absolute coefficients associated with each factor. In total, 16 overlapping gene sets have been identified with a significant coefficient in at least one condition (Additional File 3). The sets have been grouped based on the similarity of associated coefficients across all conditions into eight clusters (Figure 2): 1-histidine and glycine metabolism; 2 - sulfate assimilation; 3 - general stress response; 4 - methionine transport and metabolism; 5 - DNA damage response and repair; 6 - purine biosynthesis; 7 - protein synthesis machinery; 8 - enterochelin biosynthesis. TMP, by virtue of its anti-DHFR mechanism, triggers different starvation responses depending on the exogenous supply of respective metabolites, as evidenced from the variations in the activity of the most responsive gene sets (Figure 2A-E). Without any supplementation, the drug induced amino acid and nucleotide starvation which could be characterized by severe down-regulation of ribosomal and purine biosynthetic genes, transient up-regulation of the methionine cluster of genes, and by late and minimal up-regulation of the damage response cluster (Figure 2A). Targets of RpoS, a stress signal responsive regulator, were also significantly up-regulated in this condition (PP > 0.998), indicating that starvation for amino acids and nucleotides was followed by a general stress response in the cells. Starvation for amino acids and thymine (Figure 2B) was characterized by the down-regulation of the purine cluster and up-regulation of the methionine cluster. Addition of adenine also lowered the extent of repression of genes encoding translation proteins as compared to TMP alone. Starvation for thymine and purines (Figure 2C), on the other hand, resulted in relative down-regulation of the methionine and sulfate assimilation clusters and in an up-regulation of glycine cleavage pathway as a response to added glycine, and in a moderate, more pronounced than in the TMP alone condition, but late up-regulation of the DNA damage genes. Histidine biosynthesis was also up-regulated relative to the conditions without AA, which is an expected response to external methionine [25]. When cells were starved only for thymine (Figure 2D), three clusters were characteristically up-regulated: DNA damage, protein and enterochelin synthesis. Other gene clusters in this condition were either unaffected or down-regulated. Addition of thymine along with other supplements (Figure 2E) prevented the inductions observed in the thymine starvation. However, thymine supplementation had no effect on the activity of genes controlling purine biosynthesis, sulfate assimilation, as well as amino acid transport and metabolism. Thus thymine counteracted the antibacterial effect of TMP and suppressed DNA damage response and transcriptional over-drive of genes involved in protein synthesis. At the same time, specific regulatory responses associated with the addition of adenine or amino acids were not complemented by the nitrogen base.

### Identification of a transcriptional signature associated with lethality

**Figure 3:**
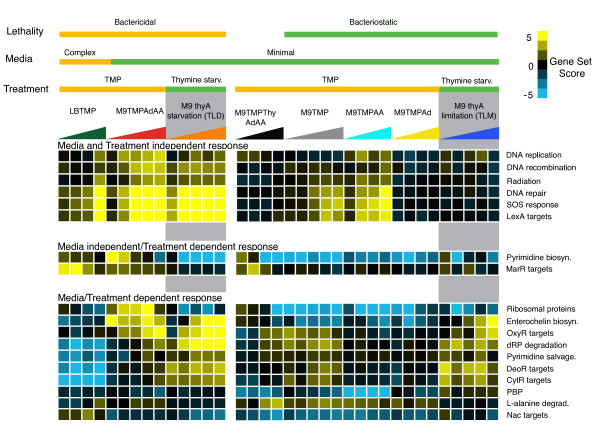
**SOS response is uniquely associated with thymineless induced lethality across treatment regimens and growth conditions**. Heatmap of gene set scores associated with TMP treatment and thymineless death (TLD)[37]. Blue and yellow color refers to down-regulation and up-regulation of gene sets respectively. Condition labels are as described in Table 1. dRP: deoxyribosephosphate; SS: supercoiling; PBP: periplasmic binding proteins.

The analysis presented in the above section supports the notion that TMP regulatory effects can be complemented by different metabolic supplements. Since, on the other hand, supplementation regimes are known to elicit different phenotypic outcomes of the drug treatment, it follows, from the transitivity principle, that the regulatory effects should be associated with the outcomes. However, since the above analysis included only one bactericidal condition (M9TMPAdAA), we chose to boost the power of our association analysis by adding another bactericidal condition: TMP treatment in LB. To determine which pathway responses were most associated with the phenotype of rapid loss of viability, we classified the gene set scores according to the lethality phenotypes, i.e. large (>50% of the treated population) loss of viability in M9TMPAdAA and LBTMP vs. no or minimal loss (< 10%) in M9TMPThyAdAA, M9TMP, M9TMPAd and M9TMPAA (Figure 3). The top gene sets associated with the phenotype were related to DNA damage response (partially overlapping Multifun categories: DNA repair and SOS response and targets of LexA; FDR < 0.01%; Additional Files 4, 5) and DNA recombination, which were up-regulated in the bactericidal conditions as compared to no change in the static conditions. Other gene sets in the top category comprised of ribosomal proteins, targets of MarR (multiple antibiotic resistance response), enterochelin biosynthesis, OxyR targets and pyrimidine biosynthesis (up in cidal, down or no change in static; "TMP treatment" in Figure 3, Additional Files 4, 5). This was in contrast with genes in deoxyribose nucleotide salvage pathways (including targets of CytR and DeoR) which were down-regulated in cidal conditions compared to the static. In particular, the salvage pathways were strongly down-regulated in complex LB medium. Iron uptake associated enterochelin biosynthesis, periplasmic binding proteins and targets of nitrogen assimilation regulator Nac were relatively down-regulated in the static conditions compared to cidal. Since the significance of the association depends on the scale of the calculated set scores, we also implemented a non-parametric classification method based on receiver-operator characteristics (ROC) to identify the specificity and sensitivity of phenotype prediction (AUC score) based on individual set scores. When top ranked gene sets from both methods, based on FDR for association and the AUC value for ROC-based classification, were combined by averaging the top gene set ranks, there was a significant overlap between the gene sets ranked by both methods (Spearman rank correlation over all gene sets = 0.96, p-value < 10^-16 ^; Additional File 6). When the responses of these top pathways were compared across all examined conditions (cidal and static conditions and complex and minimal media), transcription of only three non-redundant sets - SOS and related DNA damage gene classes, pyrimidine biosynthesis and MarR targets - was affected by the drug in the same direction, i.e., these pathways were up-regulated in each TMP-induced bactericidal condition ("TMP Treatment" in Figure 3). Directionality of transcriptional responses of the rest of the pathways depended on the media. For example, salvage pathways of deoxyribonucleotides, which were significantly associated with lethality, were down-regulated in LBTMP, but were not affected in M9TMPAdAA and were only moderately up-regulated in the static conditions. Similarly, ribosomal proteins were moderately down-regulated in LBTMP but strongly up-regulated in M9TMPAdAA. Thus, SOS response, pyrimidine biosynthesis and MarR targets, which controls transcription of genes involved in multidrug efflux, form the media independent signature responses associated with the bactericidal effect of the antibiotic.

To further compare transcriptional sensitivities of these gene sets, we scored their responses in another thymine starvation condition, triggered by thymine withdrawal from a *thyA *strain ("Thymine starvation" in Figure 3). We observed that the SOS response followed the up-regulation trend following the treatment, while pyrimidine biosynthesis was down-regulated and MarR targets were not affected. Thus, we propose that the induction of the DNA damage response is the only transcriptional effect associated with thymineless death phenomenon observed across a variety of experimental conditions including thymine starvation of auxotrophic mutants, starvation of dTMP using TMP in the presence of supplements or using TMP in complex growth media; whereas up-regulation of pyrimidine synthetic genes and MarR targets is associated with lethality only in TMP treatments. Of note, when we examined a compendium of expression profiles for activity of the MarR regulon, we observed that other top conditions in which MarR targets were up-regulated were treatments with antibacterials and antibiotics including Norfloxacin, Streptomycin, Kanamycin, Indole-acrylate and Chloramphenicol (data not shown). Since all of these treatments were carried out in LB, and the current identification of the MarR signature was driven by the LBTMP condition, it is possible that de-repression of MarR targets is a result of interaction(s) between yet unidentified factors associated with growth in a rich medium and the generic antibiotic induced stress.

We previously reported that the DNA damage response was the primary transcriptional response that separated thymine starvation leading to thymineless death (TLD) in *thyA *auxotrophs from the thymine limitation (TLM) wherein cells were supplied with suboptimal amounts of thymine [37]. Here, we showed that the SOS response was also significantly associated with cell death by TMP in minimal medium supplemented with folate metabolites or in complex medium. The genes involved in SOS response were observed to be significantly induced in bactericidal conditions as compared to static conditions after 30 minutes of treatment (One-sided t-test p-value = 0.004) and at later time points. No loss of viability was observed in cidal conditions in either media at this time point, indicating that DNA damage precedes viability loss and thus may be causative to cell lethality.

### Genome-wide genetic screen for TMP susceptibility

To complement a genome-wide transcriptional survey, we assessed the relative fitness contribution of individual gene products under TMP stress by using a forward genetic screen. To do so, we used the Keio collection of *Escherichia coli *BW25113 knock-out strains affecting 3985 individual genes [32] to screen for mutants whose growth was resistant or sensitive, as compared to wild-type, to sub-lethal concentration of TMP in LB complex medium. After 24 hours of incubation, the final yield of biomass of the mutants was measured as optical density (O.D.) using a plate reader. Since the mutants were arranged in 96-well plates randomly, outliers on each plate were identified as normalized O.D. readings that deviated significantly from the average on each plate. A list of top 100 resistant and sensitive candidates was assembled from all plates and was compared to a replicate assay. The O.D. of the highest yielding mutants was on average 6-fold higher than that of the lowest mutants in this sub-lethal concentration, and were about 70% higher than the average mutant. A total of 18 and 13 gene knock-outs had reproducible high-yield (enrichment p-value < 10^-16^) and low-yield (p < 10^-6^) phenotypes, respectively, and are listed in Tables 2 and 3.

**Table 2:** List of mutants yielding high biomass in presence of TMP at sub-lethal concentrations in LB medium

Resistant knock-out candidates		
**Gene**	**Gene ID**	**Description**

moaE	b0785	Molybdopterin (MPT) synthase, large subunit; chlorate resistance; dimer of dimers with MoaD
ybhN	b0788	Function unknown
yliE	b0833	Function unknown
ymfQ	b1153	Function unknown
yobG	b1826	Function unknown
yecN	b1869	Function unknown
crr	b2417	Phosphocarrier protein for glucose of the PTS; Enzyme IIA(Glc); formerly EIII(glc)
**recJ**	b2892	Single-stranded DNA-specific exonuclease, 5'-3'
ygjT	b3088	Function unknown; induced by alkali; putative membrane transport or efflux protein
ygjU	b3089	Na+/serine (threonine) symporter
nanA	b3225	N-Acetylneuraminate lyase (aldolase)
yhiP	b3496	Function unknown
uhpT	b3666	Fosfomycin sensitivity; sugar P transport system; transport protein for hexose P's
yiiQ	b3920	Function unknown
**uvrA**	b4058	Excision nuclease subunit A; repair of UV damage to DNA; LexA regulon; binds Zn(II)
fimE	b4313	Site-specific recombinase for fimA promoter segment inversion; bias for ON to OFF phase switching
**deoC**	b4381	Deoxyribose-phosphate aldolase; deoxyriboaldolase; binds selenium
**deoA**	b4382	Thymidine phosphorylase

A total of 18 mutants, based on their relatively high biomass yield in presence of TMP, were identified as sensitive candidates. Genes whose functions are related to DNA maintenance and nucleotide salvage pathway are highlighted in bold. Genes are sorted by their Gene IDs.

**Table 3:** List of mutants yielding low biomass in presence of TMP at sub-lethal concentrations in LB medium

Sensitive knock-out candidates		
**Gene**	**Gene ID**	**Description**

**deoR**	b0840	Repressor for deo operon, nupG and tsx; binds deoxyribose-5-phosphate inducer
flgH	b1079	Flagellar synthesis, basal body L-ring protein
fliD	b1924	Hook-associated protein 2, axial family; flagellar regulon
fliI	b1941	Cytoplasmic membrane ATPase involved in flagellar assembly; involved in export of flagellar axial protein subunits
fliN	b1946	Flagellar switch protein
JW5799	-	
mdoH	b1049	Membrane glycosyltransferase
**tdk**	b1238	Deoxythymidine kinase
ycbC	b0920	Function unknown
yciM	b1280	Function unknown
ydhA	b1639	Function unknown
yfgE (hda)	b2496	Required for regulatory inactivation of DnaA; multicopy suppressor of dnaN(ts)
alaS	b2697	Alanine--tRNA ligase

A total of 13 mutants, based on their relatively low yields, were identified as sensitive candidates. Genes whose functions are related to DNA maintenance and nucleotide salvage pathway are highlighted in bold. Genes are sorted by their Gene IDs.

The gene *tdk *encodes thymidine kinase, which converts thymidine to thymidine monophosphate dTMP (Figure 1). In the absence of *tdk*, cells exclusively rely on tetrahydrofolate dependent conversion of dUMP to dTMP, which is inhibited during TMP treatment. Cells without *tdk *should be therefore unable to grow, and indeed a *tdk *knock-out was identified as a sensitive mutant in the assay. This was verified by the observation of rapid loss of viability of *tdk *mutant after 50 μg/ml TMP addition in liquid culture (data not shown). Other mutants imparting sensitivity to TMP included several knock-outs of the genes of chemotaxis pathway (*fliD, fliL, fliN, flgH*) and of *hda*, a regulatory inhibitor of the replication initiator protein DnaA. Inactivation of Hda is a major cause of DnaA-dependent over-initiation of the chromosomal replication [38], and recent studies have proposed a link between aberrant DNA replication initiation and TLD [37,39,40]. High-yielding mutants could in principle be genuine TMP resistant mutants or strains filamenting in the presence of the drug. Among these mutants were knock-outs of *uvrA*, a nuclease component of the nucleotide excision repair, and of *recJ*, a single strand DNA exonuclease. A loss of *recJ *function was reported to alleviate the lethal effect of the thymine starvation [37,39,41], which is believed to be the primary bactericidal mechanism of TMP. Knock-outs of two of the genes involved in deoxypyrimidine nucleotide salvage pathway, *deoC *and *deoA*, were found to be among resistant candidates, whereas a knock-out of the repressor of the *deoCABD *operon, *deoR*, was found to be more susceptible to TMP. *deoC*, *deoA *and *deoR *mutants grew in LB without the drug as well as their isogenic parental strain. Of note, the *deoCABD *operon was down-regulated during LBTMP treatment and was among the top down-regulated gene sets/pathways in the condition. This enrichment of a single metabolic pathway and its regulator in the TMP phenotypic screen along with its transcriptional signature, and given the central role of the pathway in the conversion of thymidine and uridine nucleosides into nucleotides, led us to investigate these genes in more detail.

**Figure 4:**
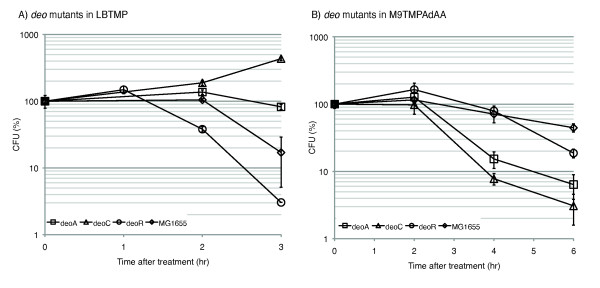
**Viability phenotypes of *deo^- ^*and *thyA^-^deo^- ^*mutants in LB and M9 media**. A) Mutants in *deoC *(Δ)*, deoA *(□) and *deoR *(○) and wild-type *E. coli *MG1655 (◇) were treated with 50 μg/ml of TMP in LB medium. Viability was tracked by counting CFU's (on LB plates after 16-hr incubation at 37°C) in the cultures sampled at 1, 2 and 3 hours of the treatment. B) In minimal media with supplements M9TMPAdAA.

The *deo *operon consists of genes encoding deoxyribose-5-phosphate aldolase (Dra/DeoC), thymidine phoshphorylase (Tpp/DeoA), pentophoshomutase (DeoB) and purine nucleotide phosphorylase (Pnp/DeoD). DeoA catalyzes the reversible interconversion between thymine and thymidine and between uracil and deoxyuridine. The deoxyribose sugar donor is deoxyribose-1-phosphate, which is converted to deoxyribose-5-phosphate (dR5P) by DeoB, and which is then degraded to glycolytic intermediates by DeoC. The operon is under negative control by DeoR. In the presence of metabolic intermediate dR5P, the *deo *operon is derepressed, leading to increased activity of the salvage pathway [42,43]. The knock-out mutations in the *deo *genes were transferred into MG1655 background by P1 transduction (Methods). First we examined if the growth phenotype of *deo *mutants translated into viability phenotypes in LB medium (Figure 4A). Viability of exponentially grown wild-type *E. coli *MG1655 treated with TMP at 50 μg/ml could be described by a typical by-phasic curve: a prolonged (120 min) lag followed by an exponential killing phase, with 17% of initial colony forming units (CFU) remaining after 3 hours of treatment. The cell mass (O.D.) typically increased 2-4 fold due to cell filamentation (Additional File 7). In comparison, MG1655 *deoA^- ^*culture retained 83% of the pre-treatment CFU's at the same time, and a *deoC^- ^*culture grew more than 400% of its original CFU's indicating nearly complete masking of the TMP effect. In contrast, a *deoR *knock-out sensitized this strain to the drug, resulting in 97% killing after 3 hours. In a complex medium, bacteria can access extracellular nucleotides and other precursors. It has been shown that the effect of TMP depends on the presence of these precursors in the medium [44]. To check for phenotypes of the *deo *mutants in a defined minimal medium, we grew cells in M9 supplemented with glucose at 0.4% and each of the folate dependent metabolites methionine, glycine and adenosine at 50 μg/ml. Upon treatment with TMP (50 μg/ml), the *deo *mutants exhibited the phenotypes opposite to those observed in LB, i.e. *deoC*^- ^and *deoA*^- ^mutants were much more sensitive (6.3% and 3.1% surviving CFU, respectively) after 3 hours of treatment compared to wild-type (44%), whereas *deoR*^- ^showed an intermediate phenotype with 18% survival (Figure 4B, Additional File 7). Addition of thymine (thymidine for *deoA^-^*) rescued these mutants from TMP. In both conditions, loss of CFU's was accompanied by cell filamentation, indicating a block in cell division (data not shown). In this assay, addition of TMP appeared to have produced a phenocopy of inactivation of thymidylate synthase, ThyA, which is required for conversion of dUMP to dTMP, by starving it for the essential methyl group donor, CH_2_-THF.

**Figure 5:**
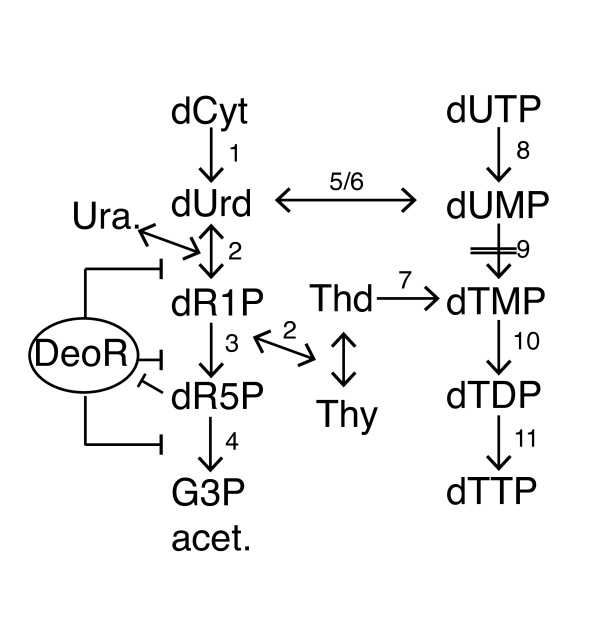
**Schematics of a pyrimidine salvage pathway**. Model of deoxyribose nucleotide salvage pathway. Double dashed line indicates block in a pathway due to gene deletion or in case of pathway 9 (dUMP → dTMP), block in pathway due to starvation of cells for essential cofactor tetrahydrofolate (not shown) by TMP treatment. Thymine and thymidine represent external metabolites transported from the media. Genes associated with pathways: 1 - cdd; 2 - deoA; 3 - deoB; 4 - deoC; 5 - tdk; 6 - yjjG; 7 - tdk; 8 - nudI/dut; 9 - thyA; 10 - tmk; 11 - ndk. Abbreviations: dCyt: deoxycytidine; dUrd.: deoxyuridine; Ura. - uracil; dR1P - deoxyribose-1-phosphate; dR5P: deoxyribose-5-phosphate; G3P/acet.: glyceraldehyde-3-phophate/acetaldehyde; Thd - thymidine (external); Thy - thymine (external).

Thus the effect of the *deo *mutations on TMP activity falls into two categories. One - affects thymidine and thymidine/thymine scavenging from the reach medium. In this category, *deoC^- ^*and *deoA^- ^*mutant cells were less susceptible to the drug than the wild type under the same treatment conditions by improving anabolism of the nucleoside/nucleotide, and *deoR^- ^*made cells more susceptible to TMP by de-repressing the thymi(di)ne catabolism. Another - affects catabolism of dUTP/dUMP/dU. In this category, both *deoC^- ^*and *deoA^- ^*mutants were more susceptible to the drug action in the minimal medium, where bacteria "relies" solely on *de novo *pyrimidine synthesis. In more detail, the phenotypes of the *deo *operon may be understood by examining the nucleotide salvage pathway (Figure 5). In the presence of exogenous deoxyribose pyrimidines, the pathway catabolizes the ribose sugars to form glyceraldehyde-3-phoshpate and acetaldehyde to be used in central carbon metabolism. However, during thymine starvation in *thyA*^- ^mutants and TMP treatment, the cell salvages thymine, or thymidine, from the medium using dR1P. In LBTMP condition, CH_2_-THF will eventually deplete as cells run out of folate dependent metabolites, causing dTMP (and hence dTTP) insufficiency. LB medium contains thymidine (as ascertained by the sustained growth of *thyA*^-^*deoA*^- ^and *thyA*^- ^strains in the medium), which is utilized. However, since thymidine is catabolized to dR1P and its catabolic products, the equilibrium of thymidine/thymine reversible conversion by thymidine phosphorylase (DeoA) should be shifted toward thymidine catabolism. Since thymidylate is catabolized, cells eventually starve for dTTP and lose viability. The *deo *operon is down-regulated in the LBTMP condition as compared to before treatment, suggesting depletion of dR5P caused by utilization of dR1P by thymine to replenish thymidine for dTTP synthesis. In the absence of functional *deoA*, cells are able to utilize all the thymidine in the medium for dTTP synthesis, since it cannot be catabolized to thymine. Furthermore, when *deoC *is mutated, not only is thymidine not catabolized, the accumulation of dR5P may also derepress *deoA *to allow the scavenging of thymine from the medium. This explains the refractory nature of the *deoC *and *deoA *mutants to TMP in LB. It is likely that once thymidine is exhausted from the medium, cells may continue on their bactericidal trajectory provided they sustain continued growth in the presence of other folate dependent metabolites, and provided TMP is effective in inhibiting DHFR. However, when the operon is completely derepressed due to the absence of *deoR*, the cells fail to repress the catabolism of thymidine and they may run out of thymidine quicker than wild-type strain in LB explaining the sensitive phenotype.

In minimal medium supplemented with amino acids and purine, cells cannot utilize exogenous thymine or thymidine like in LB. dTTP depletion during thymine starvation induces the ribonucleoside-diphosphate reductases, which increases flux through the dTTP biosynthetic pathway and causes an accumulation of dCTP and dUMP [45,46]. In M9TMPAdAA condition, we see an up-regulation of the *nrdAB*, genes (log-ratio > 0.5) responsible for the reduction of pyrimidine ribonucleotides to dUTP, indicating that similar conditions may exist during this treatment. The excess accumulated dUTP can then get incorporated into DNA or can be degraded to dUMP and further to deoxyuridine and dR1P via the dUMP phosphatase YjjG [42,43,47]. During thymine starvation, excess ribose sugar is secreted into the medium, particularly in *deoC*^- ^mutants [48]. In a *deoA*^- ^mutant, the cells lack the ability to degrade deoxyuridine and this may lead to the increased accumulation of dUMP and dUTP in the cells, possibly due to the conversion of the accumulated deoxyuridine to dUMP via thymidine kinase [49].

Similarly, in *deoC*^- ^mutant, dUMP cannot be catabolized leading to dUTP accumulation. The slight difference in sensitivity in *deoA *and *deoC *to TMP (Figure 4B) could be attributed to the activity of uridine phosphorylase (Udp) which also degrades deoxyuridine at much lower rate than DeoA [50]. Finally, the *deoR*^- ^mutant should be similar to the wild-type since in the wild-type, the *deo *operon is derepressed due to accumulation of dR5P, and the loss of viability in *deoR *is similar to that in the wild-type strain.

In the current study, we observed that the kinetics of killing by TMP was similar to that by thymine starvation: 1-2 hour lag is followed by a relatively rapid loss of viability. Moreover, induction of the SOS response in both conditions occurred in the first half of the lag stage, indicating that DNA becomes damaged long before the cells loose their ability to divide. The duration of the lag stage can in principle be determined by two factors: 1- the time it takes for dTTP to drop below a certain level to cause replication fork arrest; 2 - the time it takes for DNA damage to become irreversible. While the first factor can be postulated *a priori*; the second is derived from the observation that the loss of RecA or RecBC function shortens or eliminates the lag [41], suggesting that double-strand break repair pathway has a capacity to reverse some effects of the starvation. It is likely that the shortening of the lag in a *deoR*^- ^mutant in the rich medium was due to faster depletion of the dTTP pool, and the phenotype of *deoC*^- ^and *deoA*^- ^mutants in the rich medium can be viewed as an extension of the lag stage due to the pool maintenance in the mutant cells effectively scavenging thymi(di)ne. When both dTTP and dUTP pools become fully exhausted, DNA damage becomes persistent and its processing by some repair enzymes makes the damage irreversible. Cells from this stage in the thymineless death pathway cannot be recovered and that accounts for a rapid decline in viability. In support of this view, mutants that can't process initial DNA damage, e.g., *recF^-^*, loose viability at a much-reduced rate following the lag [39,41].

It appears that the primary factor in establishing the rate of starvation-induced killing is a number of chromosomal replication events in a bacterial population. The higher the rate of initiation and the greater the number of growing replication forks per chromosome, the higher the probability of the irreversible chromosomal collapse [40]. In principle, if the dTTP pool is efficiently replenished with dUTP, that would prevent replication fork arrest (at least in the current replication cycle) and thus would reduce the probability of the subsequent chromosomal collapse. Such replenishment does not happen under conventional thymine starvation in *thyA *auxotrophs because of the activity of dUTPase, which efficiently hydrolyzes dUTP to dUMP and PPi [51] and because of the yjjG-deo pathway, which catabolizes excess dUMP and dU [42,43,47]. That is probably why the thymineless death triggered by thymine starvation in thymidylate auxotrophs is not affected by the activity of uracil-N-glycosyase, the first enzyme in the uracil base excision pathway [41,52]: although uracil may be occasionally found in DNA from some mutant TLD cells, its post-replicative excision would not matter much since the chromosome would be already on its way toward collapse from the fork arrest, i.e., for uracil to be incorporated in a part of DNA, the replication fork must traverse that part of DNA in the template, incorporation of U signifies exhaustion of dTTP and since dUTP are readily hydrolyzed, the fork that started incorporating U's is subject to an eminent arrest. As a result, the kinetics of the viability loss would be determined by the frequency of fork arrests and not by the frequency of other DNA lesions.

However, in the conditions that maybe prone to a slower exhaustion of the dTTP pool, due to indirect or limited inhibitory effect on the thymidylate synthase and/or significant increase of dUTP over dTTP, incorporation of uracil may become a factor in determining the outcome of dTTP starvation. For example, chromosomes that are fully replicated in the presence of sufficient dUTP may eventually collapse as a result of uracil excision repair, which could be initiated but not completed in the absence of dTTP or dUTP. That appears to be the case in yeast treated with the antifolate inhibitor aminopterin [53] and in animal cells treated with the antifolate drug methotrexate [54]. We argue that at least in *deoC*^- ^and *deoA*^- ^cultures treated with TMP, a fraction of cells can complete the ongoing rounds of DNA replication, using dUTP in combination with slowly decaying dTTP supply, and these chromosomes would collapse primarily because of uracil processing and not because of replication fork arrest. Consistent with that is the observation that the lag stage was not affected in the *deoC*^- ^and *deoA*^- ^mutants; instead, the loss of viability was relatively accelerated in these mutants - pointing at an additional source(s) of irreversible DNA damage. It is possible that TMP treatment of *E. coli*, independent of the growth medium and similar to anti-folate treatments in higher organisms, results in dUTP levels sufficient to support DNA replication. Processing uracil out of such DNA with UNG would be detrimental to the cells. This assertion is supported in part by our observation that an *ung*^- ^mutant yielded in our screen 3 times as much biomass as an average strain (data not shown) and by observations from a chemical genomics screen where *dut*^- ^and *ung*^- ^mutants showed fitness defect, while *dcd*^- ^showed fitness advantage in response to the TMP treatment [55]. Thus, it is likely that depending on the mechanism of thymine starvation (TMP versus direct thymidylate deficiency) and despite apparent similarities between them, different components of DNA metabolism may contribute to the starvation outcomes.

## Conclusion

Treatment of E. coli bacteria with the antibiotic Trimethoprim results in regulatory responses that can be associated with the drug mechanism as well as treatment conditions. Depending on the treatment condition, the drug can have bacteriostatic or bactericidal effect. The DNA damage response can be used to discriminate between the bacterial killing, which is preceded by the damage response, and the bacteriostasis, during which the damage response does not develop. The drug induces DNA damage at the beginning of the characteristic lag stage of the treatment. Mutations in the deoxyribose nucleotide salvage pathway can affect duration of the lag as well as the rate of killing. The duration of the lag is likely determined by the capacity of the cells to buffer the effect of thymine starvation caused by the drug, e.g., through dTTP pools. The rate of killing, however, is determined by the distribution of drug sensitivities across a bacterial population. The sensitivity of individual cell in turn should be determined by its stage in the replication and division cycles. We speculate that Trimethoprim, by affecting replication and possibly division cycles, makes uracil incorporation into DNA an important source of the offending lesion in the drug-induced thymine starvation.

## Authors' contributions

DS performed the genetic screen, analyzed the data and co-wrote the manuscript. ZZ performed the microarray experiments. AK conceived of the study and co-wrote the manuscript. All authors read and approved the final manuscript.

## Supplementary Material

Additional file 1 **Gene Sets**. A list of gene sets used in the analysis and individual genes that make up sets. The file contains two excel sheets: "Gene Sets" enumerates all classified sets; "Gene Set membership" classifies genes into sets: 1 - a member, 0 - not a member. Click here for file

Additional file 2 **Analysis code**. R code used for the analysis of the data and tools for retrieving individual gene set scores and generating plots. Click here for file

Additional file 3 **Coefficients of a set score linear model on individual supplements**. Set scores and set coefficients obtained from the linear model employed to assess the effects of individual supplements (as described in main text). Click here for file

Additional file 4 **Gene Sets associated with the lethality phenotype**. List of gene sets up-regulated in bacteriostatic or bactericidal conditions along with their linear model coefficients. Click here for file

Additional file 5 **Plots of gene set scores associated with the lethality phenotype**. Boxplots identifying the set score differences between static and cidal conditions. Click here for file

Additional file 6 **Comparison of non-parametric (AUC) and parametric (linear model FDR) significant tests for sets**. Scatter plot of AUC scores and False Discovery Rates (FDR) from the linear model indicating strong correlation between the two measures of association between set scores and cell phenotype. Click here for file

Additional file 7 **O.D. values of *deo *mutants after TMP treatment in M9 and LB media**. A) LBTMP treatment. Increase in O.D. (600 nm) after 50 μg/ml TMP treatment of wild-type MG1655 strain (blue), *deoA *(red), *deoC *(green) and *deoR *(purple) knock-out mutants in LB medium. TMP was added to respective cultures grown from overnight inoculum to mid-log phase in LB medium at O.D. 0.37-0.42 (Time 0 hr). B) M9TMPAdAA treatment. Increase in O.D. (600 nm) after 25 μg/ml TMP treatment of wild-type MG1655 strain (blue), *deoA *(red), *deoC *(green) and *deoR *(purple) knock-out mutants in M9 medium. TMP was added to respective cultures grown from overnight inoculum to mid-log phase in M9 medium at O.D. 0.25-0.35 (Time 0 hr). Click here for file
