# Supplementary material for: The association of DNA damage response and nucleotide level modulation with the antibacterial mechanism of the anti-folate drug Trimethoprim
Source: BMC Genomics. 2011 Nov 28;12:583. doi: 10.1186/1471-2164-12-583 (PMC3258297; doi:10.1186/1471-2164-12-583)
Supplement: Additional file 5 — Plots of gene set scores associated with the lethality phenotype. Boxplots identifying the set score differences between static and cidal conditions. [file 1471-2164-12-583-S5.PDF]

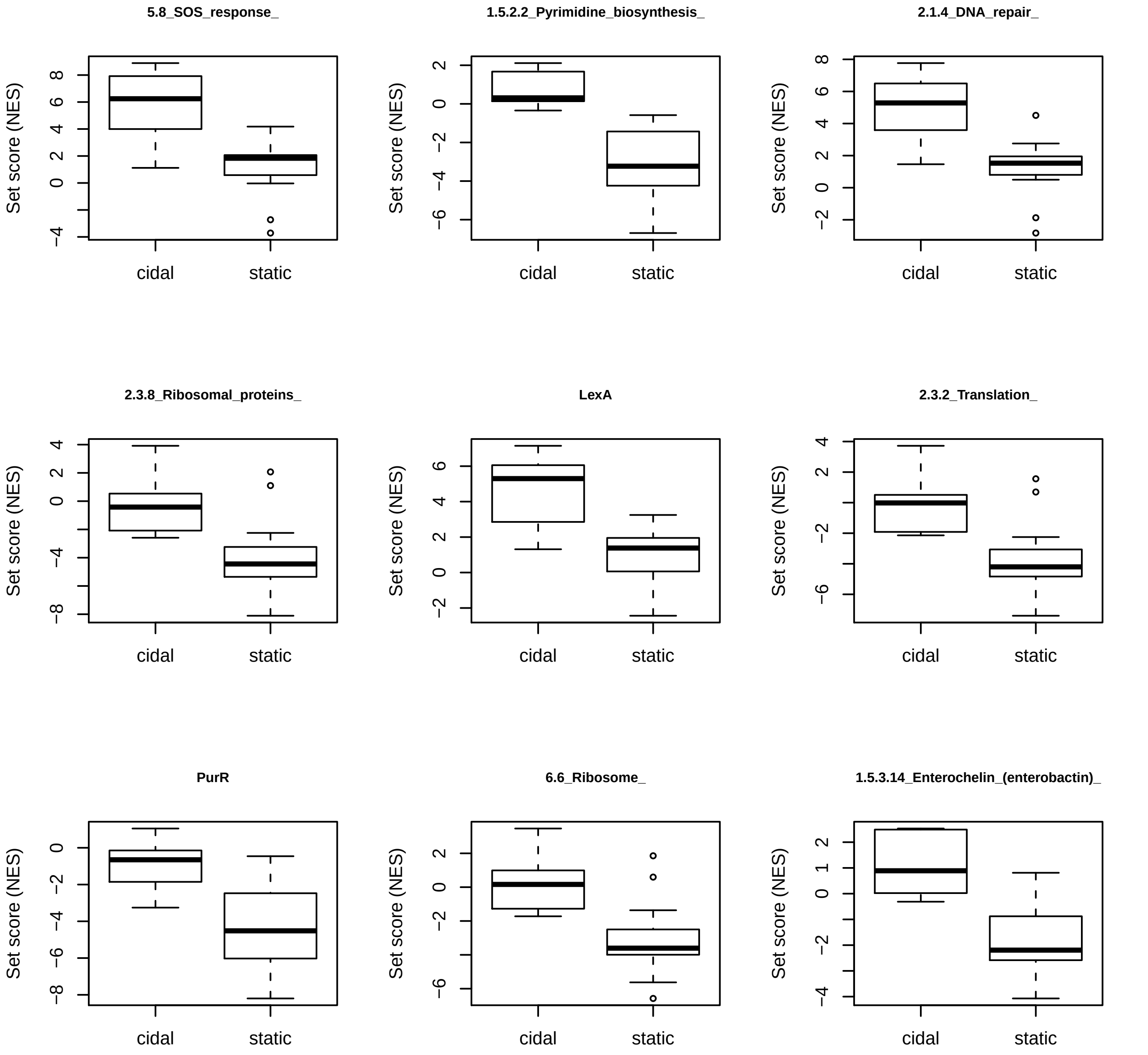
5.8_SOS_response_
1.5.2.2_Pyrimidine_biosynthesis_
2.1.4_DNA_repair_
2.3.8_Ribosomal_proteins_
LexA
2.3.2_Translation_
PurR
6.6_Ribosome_
1.5.3.14_Enterochelin_(enterobactin)_
Set score (NES)
cidal
static

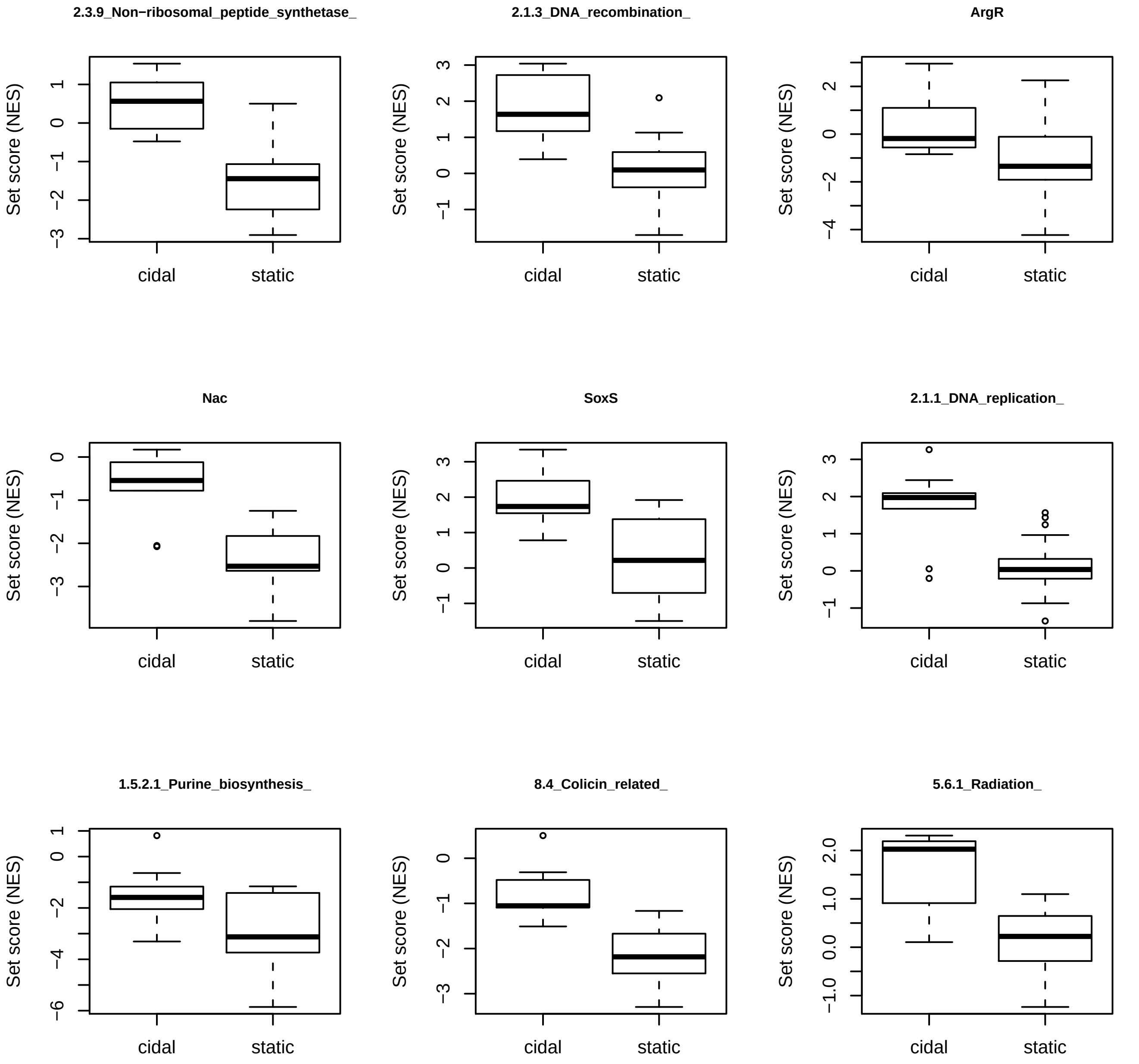
2.3.9_Non−ribosomal_peptide_synthetase_
2.1.3_DNA_recombination_
ArgR
Nac
SoxS
2.1.1_DNA_replication_
1.5.2.1_Purine_biosynthesis_
8.4_Colicin_related_
5.6.1_Radiation_
Set score (NES)
cidal
static

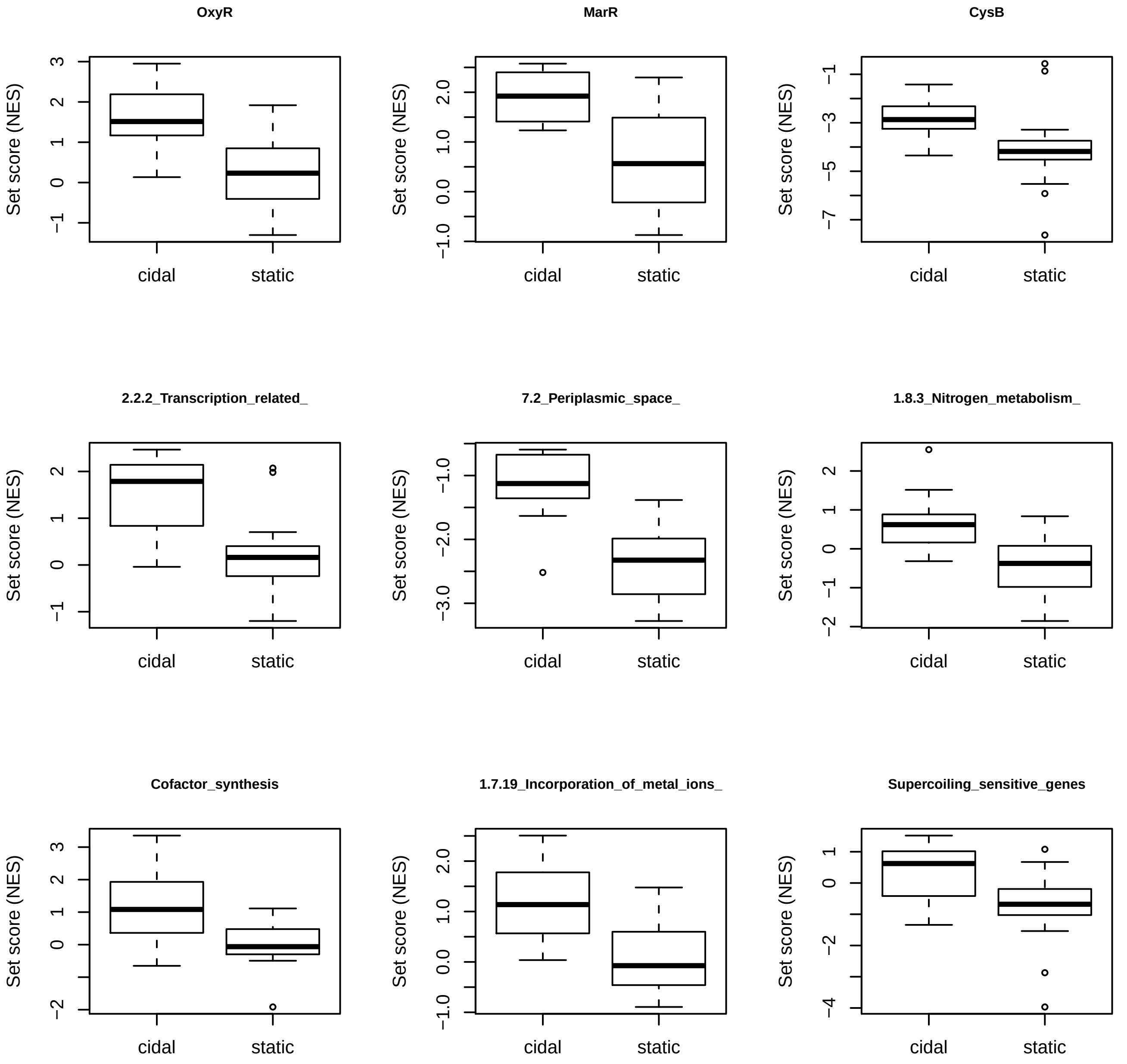

OxyR
MarR
CysB
2.2.2_Transcription_related_
7.2_Periplasmic_space_
1.8.3_Nitrogen_metabolism_
Cofactor_synthesis
1.7.19_Incorporation_of_metal_ions_
Supercoiling_sensitive_genes
Set score (NES)
cidal
static

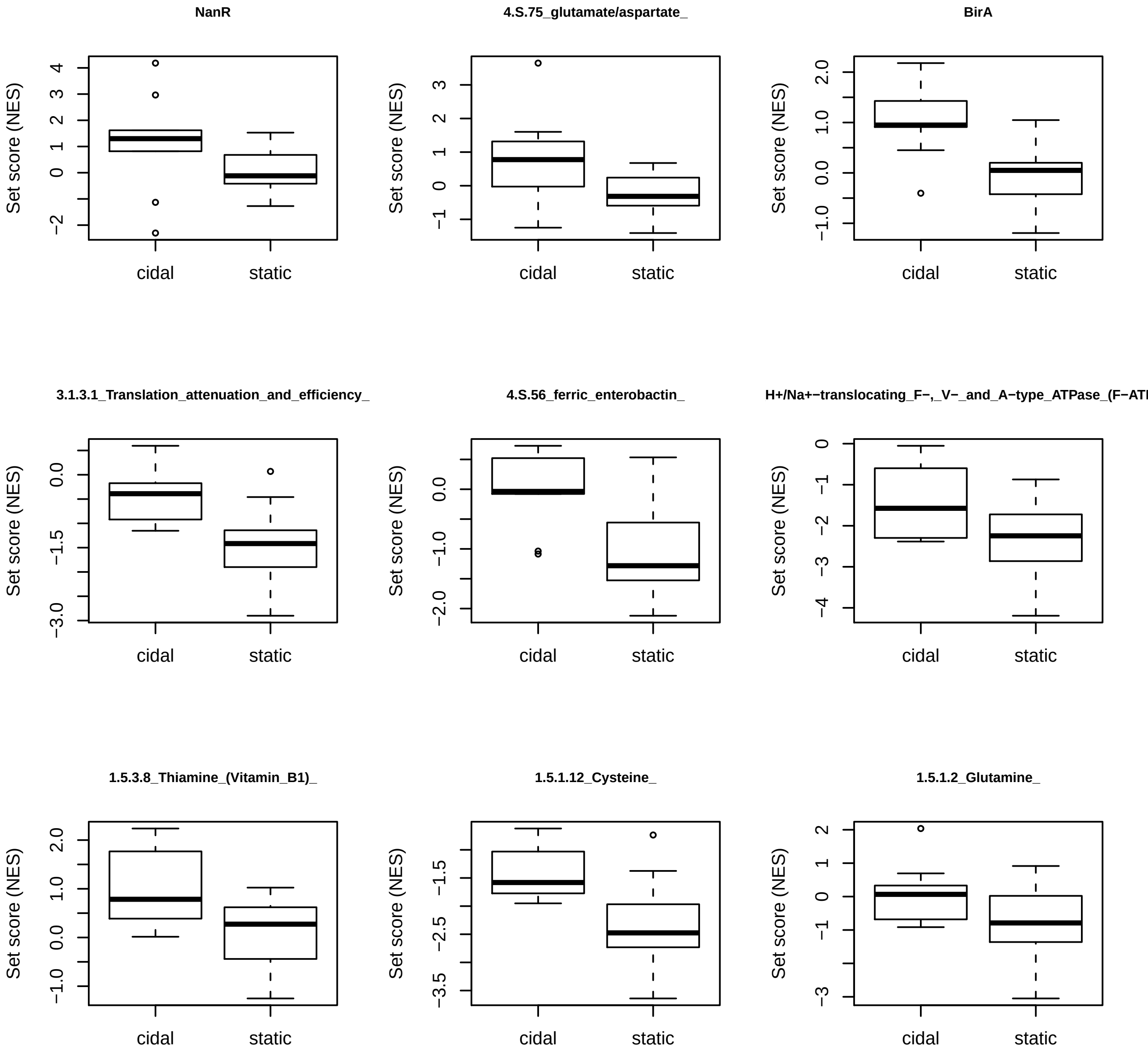
NanR
4.S.75_glutamate/aspartate_
BirA
3.1.3.1_Translation_attenuation_and_efficiency_
4.S.56_ferric_enterobactin_
H+/Na+−translocating_F−,_V−_and_A−type_ATPase_(F−AT
1.5.3.8_Thiamine_(Vitamin_B1)_
1.5.1.12_Cysteine_
1.5.1.2_Glutamine_
Set score (NES)
cidal
static

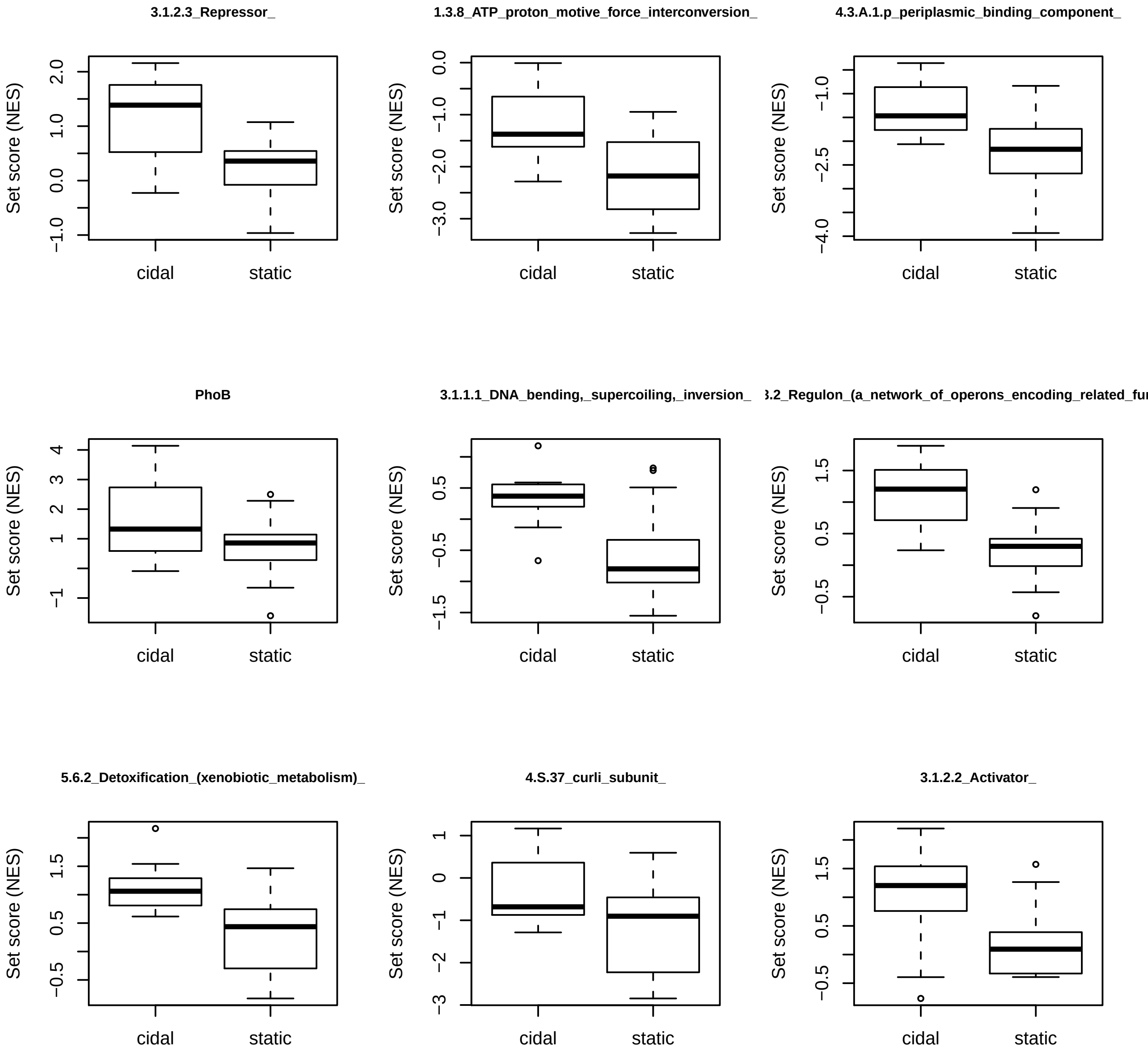

3.1.2.3_Repressor_
Set score (NES)
−1.0
0.0
1.0
2.0
cidal
static
1.3.8_ATP_proton_motive_force_interconversion_
Set score (NES)
−3.0
−2.0
−1.0
0.0
cidal
static
4.3.A.1.p_periplasmic_binding_component_
Set score (NES)
−4.0
−2.5
−1.0
cidal
static
PhoB
Set score (NES)
−1
1
2
3
4
cidal
static
3.1.1.1_DNA_bending,_supercoiling,_inversion_
Set score (NES)
−1.5
−0.5
0.5
cidal
static
.2_Regulon_(a_network_of_operons_encoding_related_fu
Set score (NES)
−0.5
0.5
1.5
cidal
static
5.6.2_Detoxification_(xenobiotic_metabolism)_
Set score (NES)
−0.5
0.5
1.5
cidal
static
4.S.37_curli_subunit_
Set score (NES)
−3
−2
−1
0
1
cidal
static
3.1.2.2_Activator_
Set score (NES)
−0.5
0.5
1.5
cidal
static

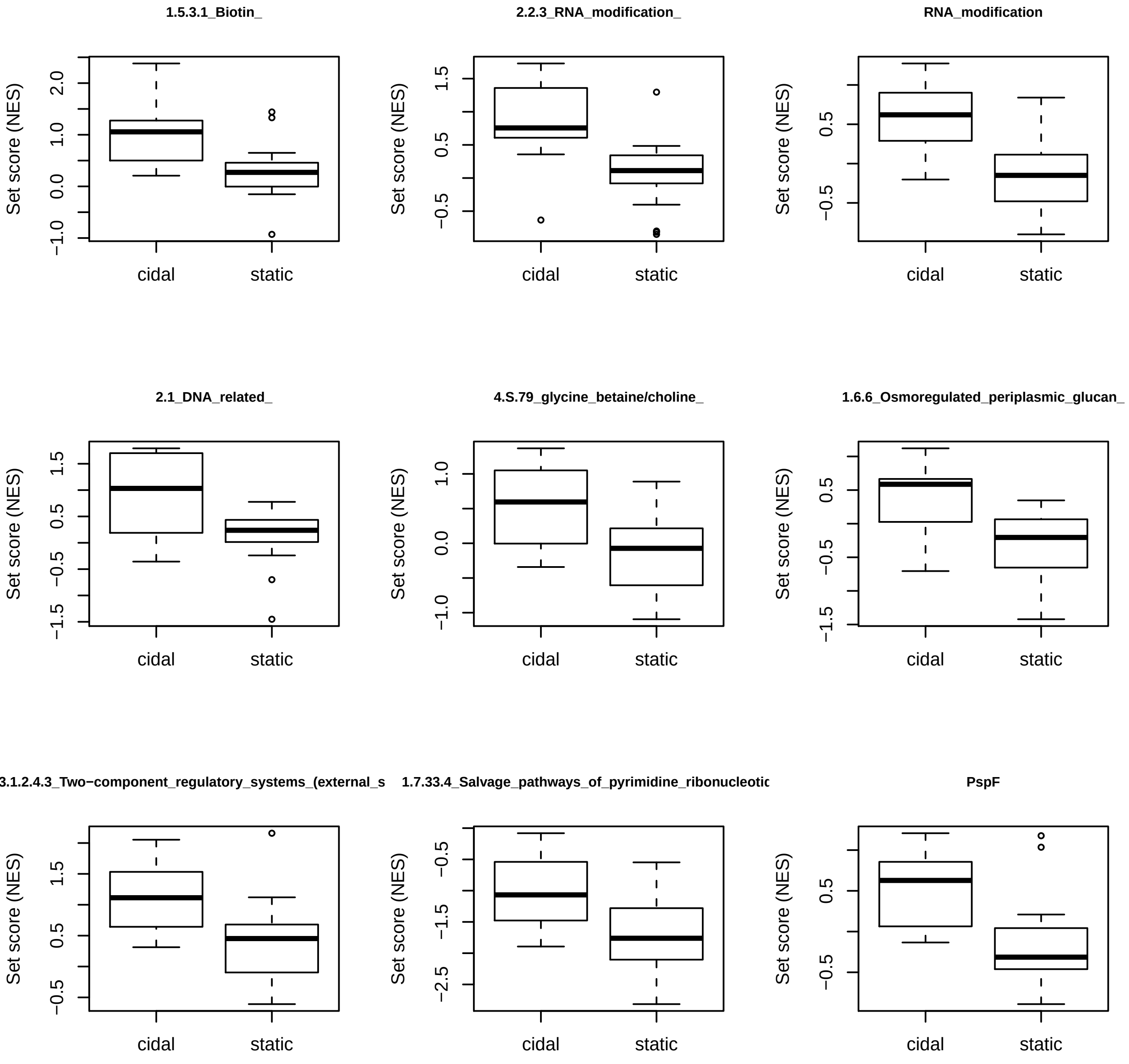
1.5.3.1_Biotin_
Set score (NES)
−1.0
0.0
1.0
2.0
cidal
static
2.2.3_RNA_modification_
Set score (NES)
−0.5
0.5
1.5
cidal
static
RNA_modification
Set score (NES)
−0.5
0.5
cidal
static
2.1_DNA_related_
Set score (NES)
−1.5
−0.5
0.5
1.5
cidal
static
4.S.79_glycine_betaine/choline_
Set score (NES)
−1.0
0.0
1.0
cidal
static
1.6.6_Osmoregulated_periplasmic_glucan_
Set score (NES)
−1.5
−0.5
0.5
cidal
static
3.1.2.4.3_Two−component_regulatory_systems_(external_s
Set score (NES)
−0.5
0.5
1.5
cidal
static
1.7.33.4_Salvage_pathways_of_pyrimidine_ribonucleotic
Set score (NES)
−2.5
−1.5
−0.5
cidal
static
PspF
Set score (NES)
−0.5
0.5
cidal
static

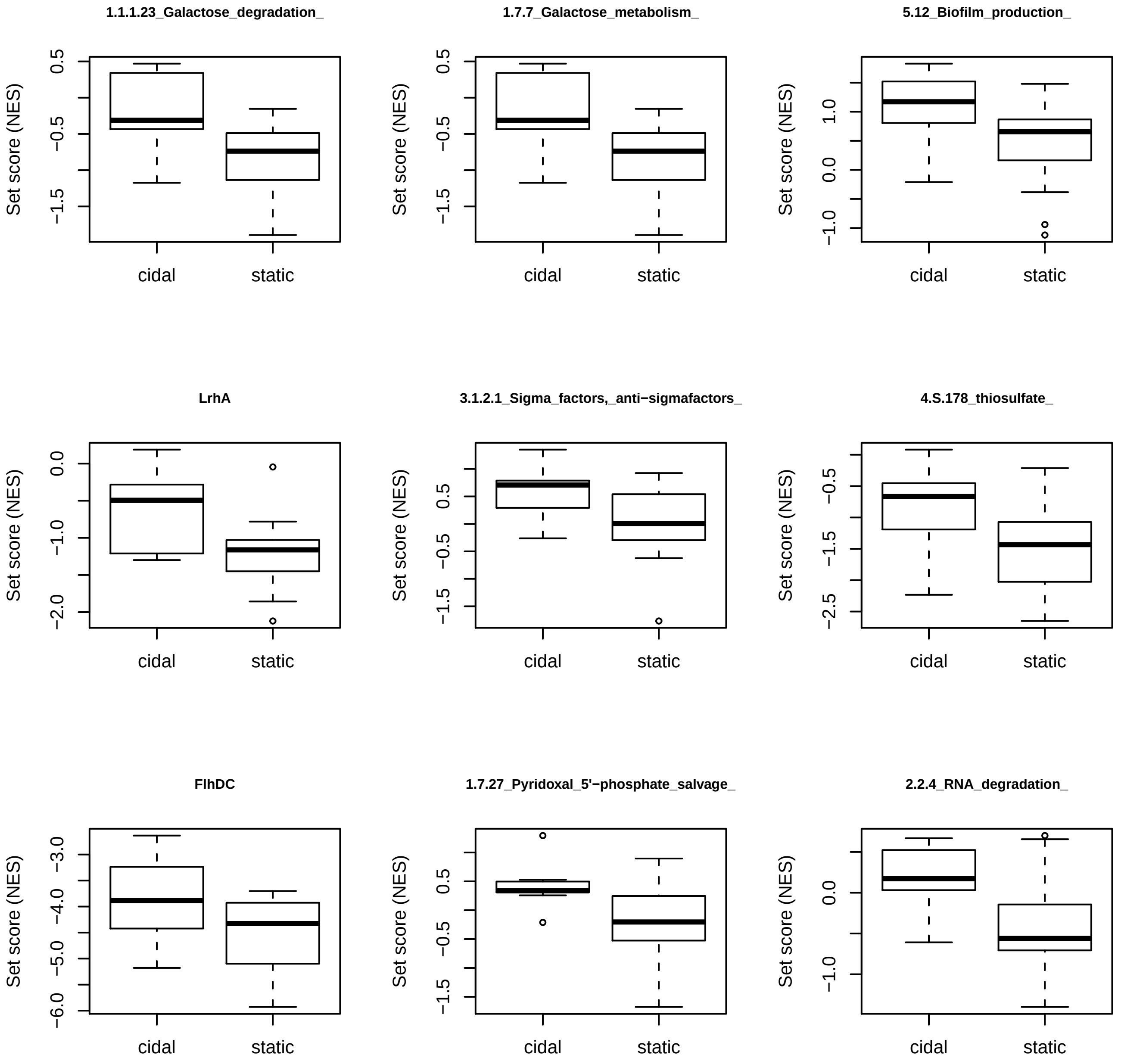
1.1.1.23_Galactose_degradation_
Set score (NES)
0.5
−0.5
−1.5
cidal
static
1.7.7_Galactose_metabolism_
Set score (NES)
0.5
−0.5
−1.5
cidal
static
5.12_Biofilm_production_
Set score (NES)
1.0
0.0
−1.0
cidal
static
LrhA
Set score (NES)
0.0
−1.0
−2.0
cidal
static
3.1.2.1_Sigma_factors,_anti−sigmafactors_
Set score (NES)
0.5
−0.5
−1.5
cidal
static
4.S.178_thiosulfate_
Set score (NES)
−0.5
−1.5
−2.5
cidal
static
FlhDC
Set score (NES)
−3.0
−4.0
−5.0
−6.0
cidal
static
1.7.27_Pyridoxal_5'−phosphate_salvage_
Set score (NES)
0.5
−0.5
−1.5
cidal
static
2.2.4_RNA_degradation_
Set score (NES)
0.0
−1.0
cidal
static

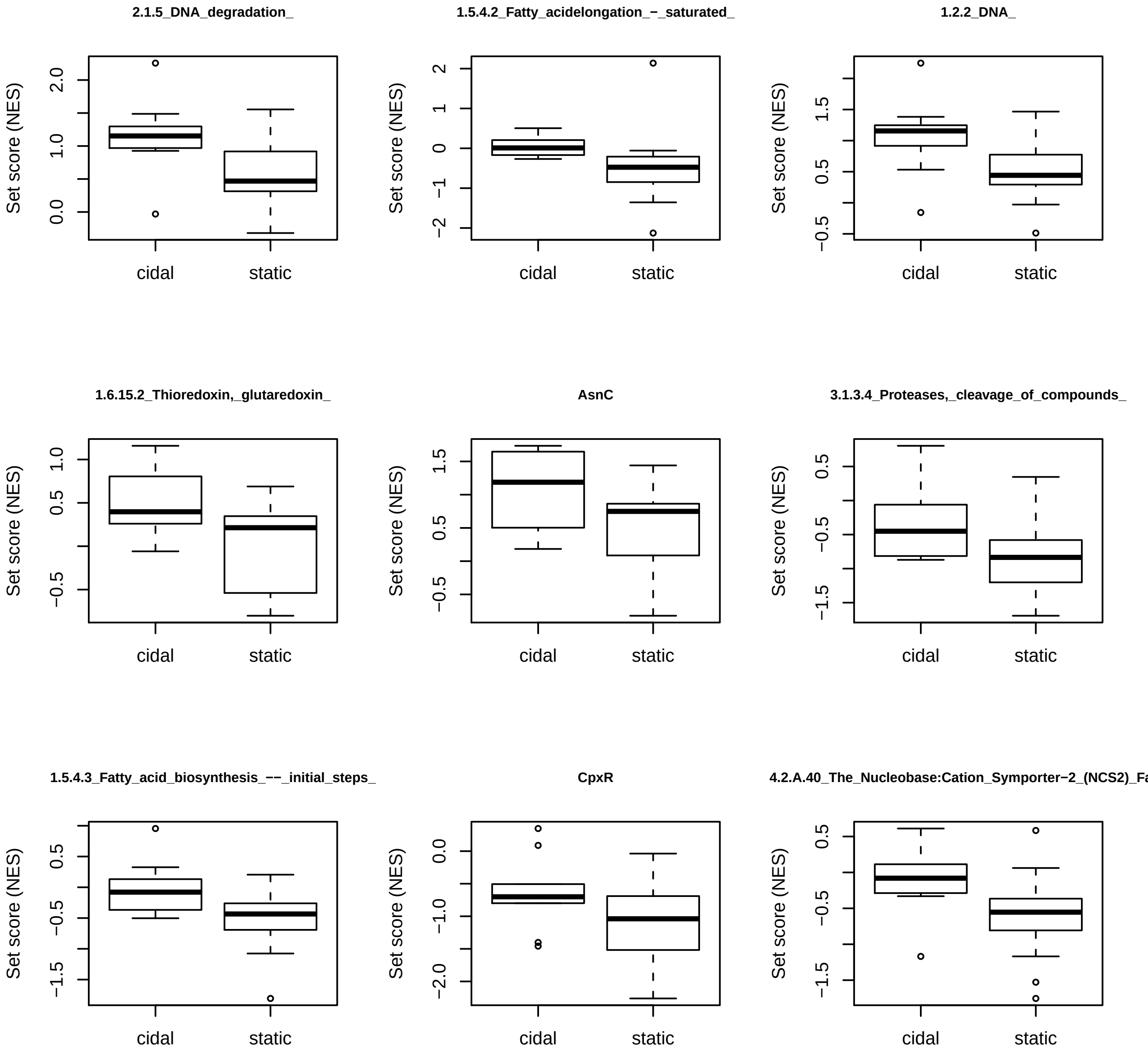

2.1.5_DNA_degradation_
1.5.4.2_Fatty_acidelongation_−_saturated_
1.2.2_DNA_
1.6.15.2_Thioredoxin,_glutaredoxin_
AsnC
3.1.3.4_Proteases,_cleavage_of_compounds_
1.5.4.3_Fatty_acid_biosynthesis_−−_initial_steps_
CpxR
4.2.A.40_The_Nucleobase:Cation_Symporter−2_(NCS2)_Fa
Set score (NES)
cidal
static

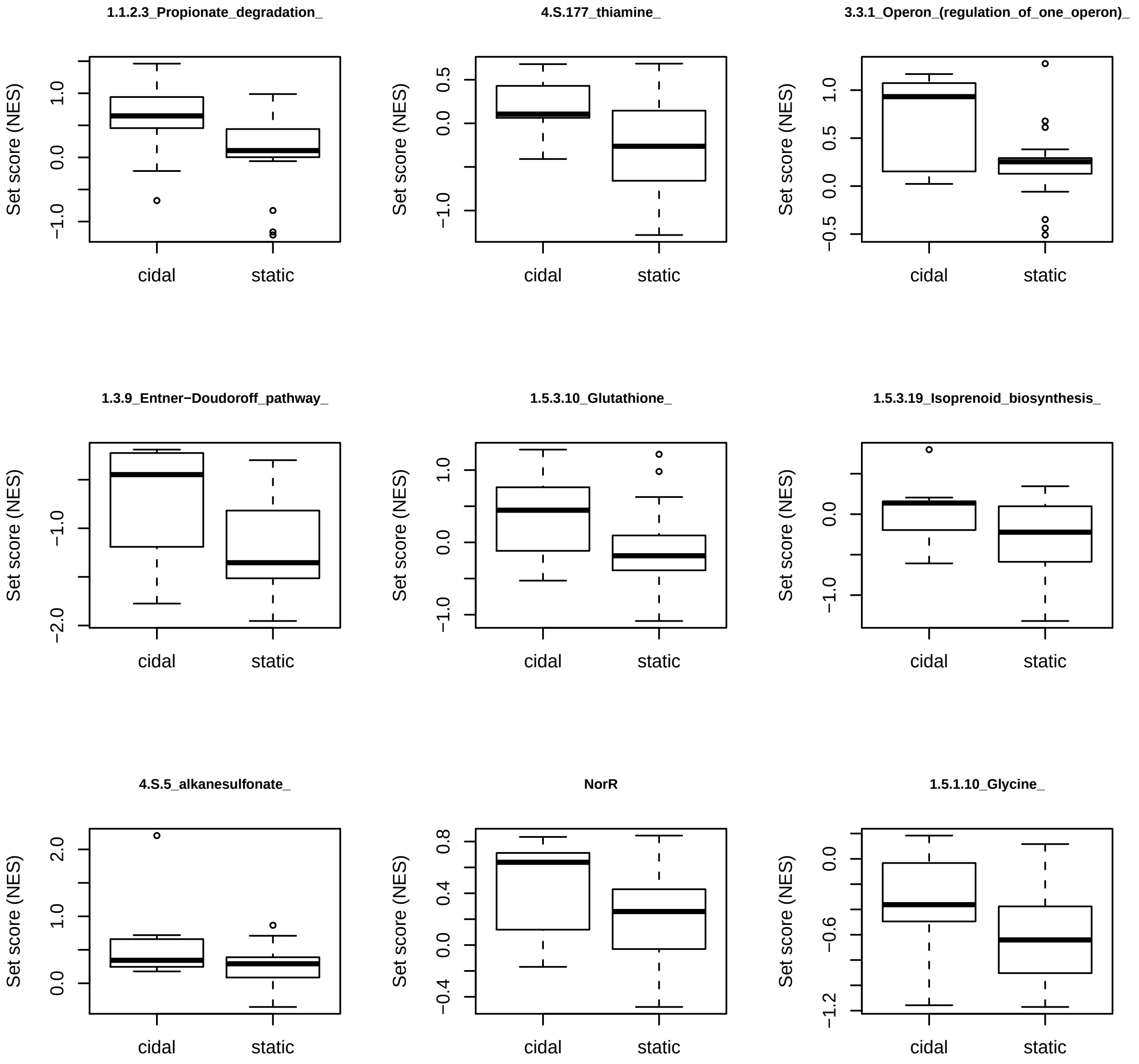
1.1.2.3_Propionate_degradation_
Set score (NES)
−1.0
0.0
1.0
cidal
static
4.S.177_thiamine_
Set score (NES)
−1.0
0.0
0.5
cidal
static
3.3.1_Operon_(regulation_of_one_operon)_
Set score (NES)
−0.5
0.0
0.5
1.0
cidal
static
1.3.9_Entner−Doudoroff_pathway_
Set score (NES)
−2.0
−1.0
cidal
static
1.5.3.10_Glutathione_
Set score (NES)
−1.0
0.0
1.0
cidal
static
1.5.3.19_Isoprenoid_biosynthesis_
Set score (NES)
−1.0
0.0
cidal
static
4.S.5_alkanesulfonate_
Set score (NES)
0.0
1.0
2.0
cidal
static
NorR
Set score (NES)
−0.4
0.0
0.4
0.8
cidal
static
1.5.1.10_Glycine_
Set score (NES)
−1.2
−0.6
0.0
cidal
static

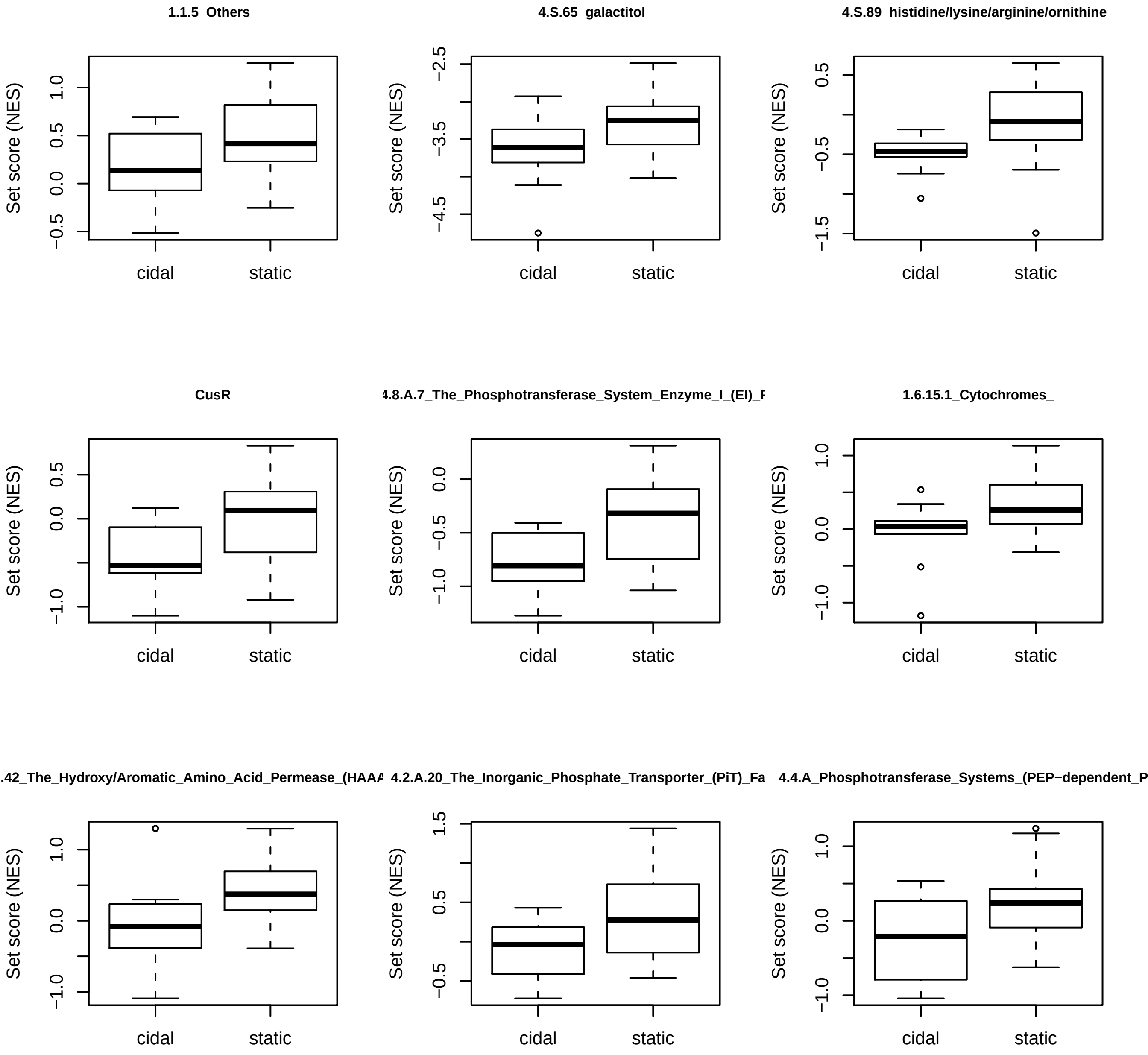

1.1.5_Others_
4.S.65_galactitol_
4.S.89_histidine/lysine/arginine/ornithine_
CusR
4.8.A.7_The_Phosphotransferase_System_Enzyme_I_(EI)_F
1.6.15.1_Cytochromes_
.42_The_Hydroxy/Aromatic_Amino_Acid_Permease_(HAAA
4.2.A.20_The_Inorganic_Phosphate_Transporter_(PiT)_Fa
4.4.A_Phosphotransferase_Systems_(PEP−dependent_P
Set score (NES)
cidal
static

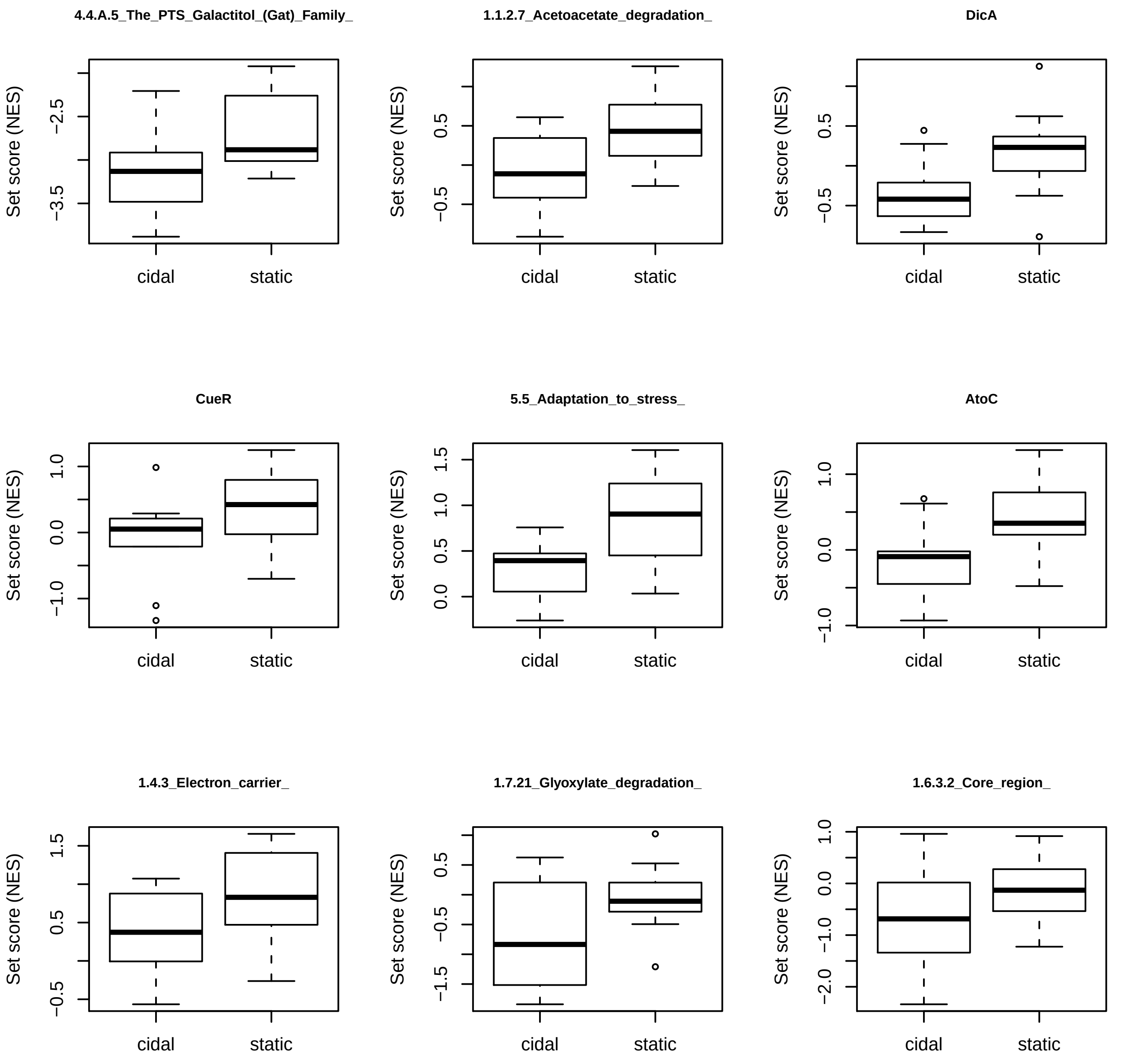

4.4.A.5_The_PTS_Galactitol_(Gat)_Family_
Set score (NES)
−2.5
−3.5
cidal
static
1.1.2.7_Acetoacetate_degradation_
Set score (NES)
0.5
−0.5
cidal
static
DicA
Set score (NES)
0.5
−0.5
cidal
static
CueR
Set score (NES)
1.0
0.0
−1.0
cidal
static
5.5_Adaptation_to_stress_
Set score (NES)
1.5
1.0
0.5
0.0
cidal
static
AtoC
Set score (NES)
1.0
0.0
−1.0
cidal
static
1.4.3_Electron_carrier_
Set score (NES)
1.5
0.5
−0.5
cidal
static
1.7.21_Glyoxylate_degradation_
Set score (NES)
0.5
−0.5
−1.5
cidal
static
1.6.3.2_Core_region_
Set score (NES)
1.0
0.0
−1.0
−2.0
cidal
static

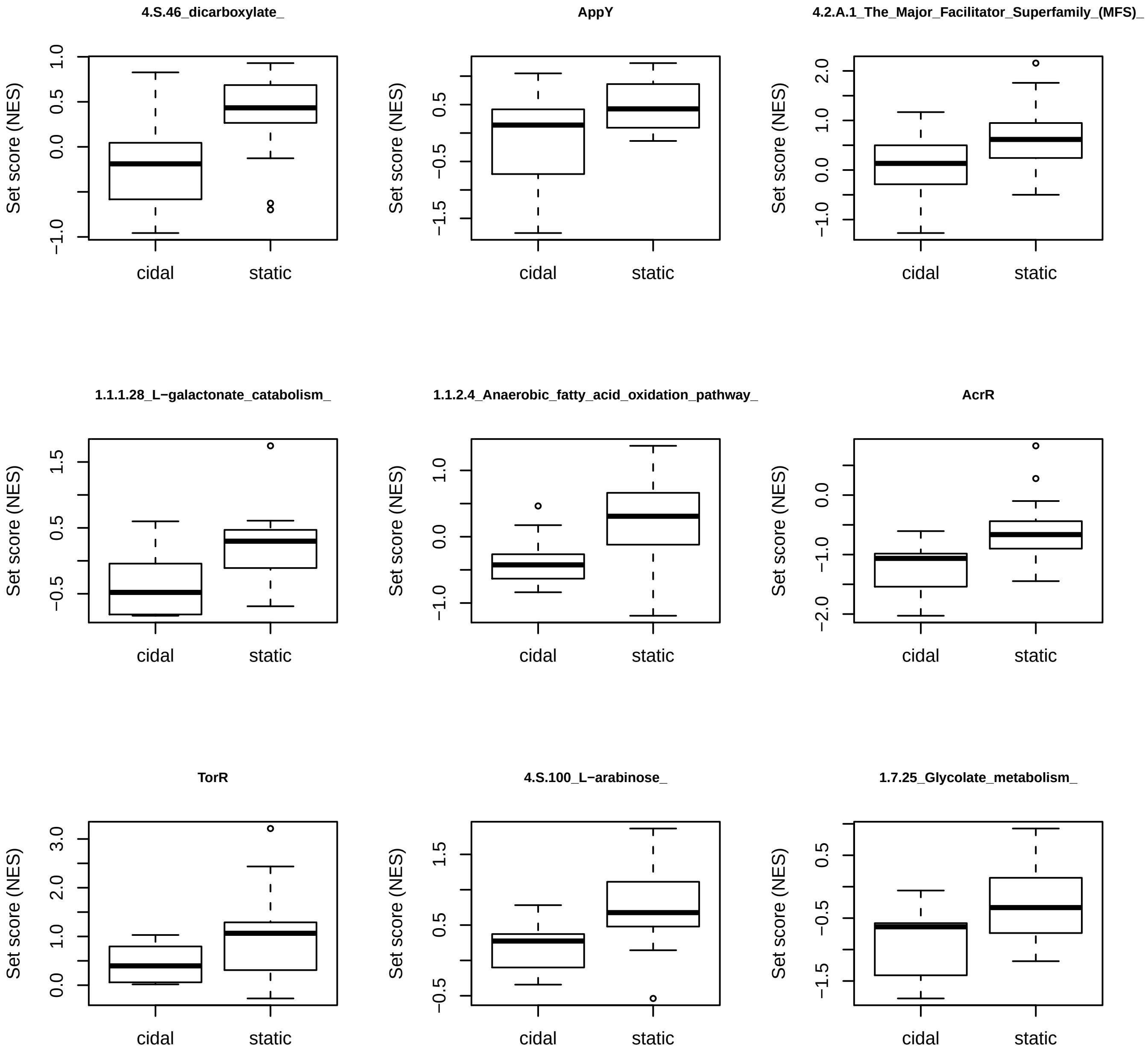
4.S.46_dicarboxylate_
AppY
4.2.A.1_The_Major_Facilitator_Superfamily_(MFS)_
1.1.1.28_L−galactonate_catabolism_
1.1.2.4_Anaerobic_fatty_acid_oxidation_pathway_
AcrR
TorR
4.S.100_L−arabinose_
1.7.25_Glycolate_metabolism_
Set score (NES)
cidal
static
−1.0
0.0
0.5
1.0
−1.5
−0.5
2.0
−0.5
1.5
−2.0
3.0

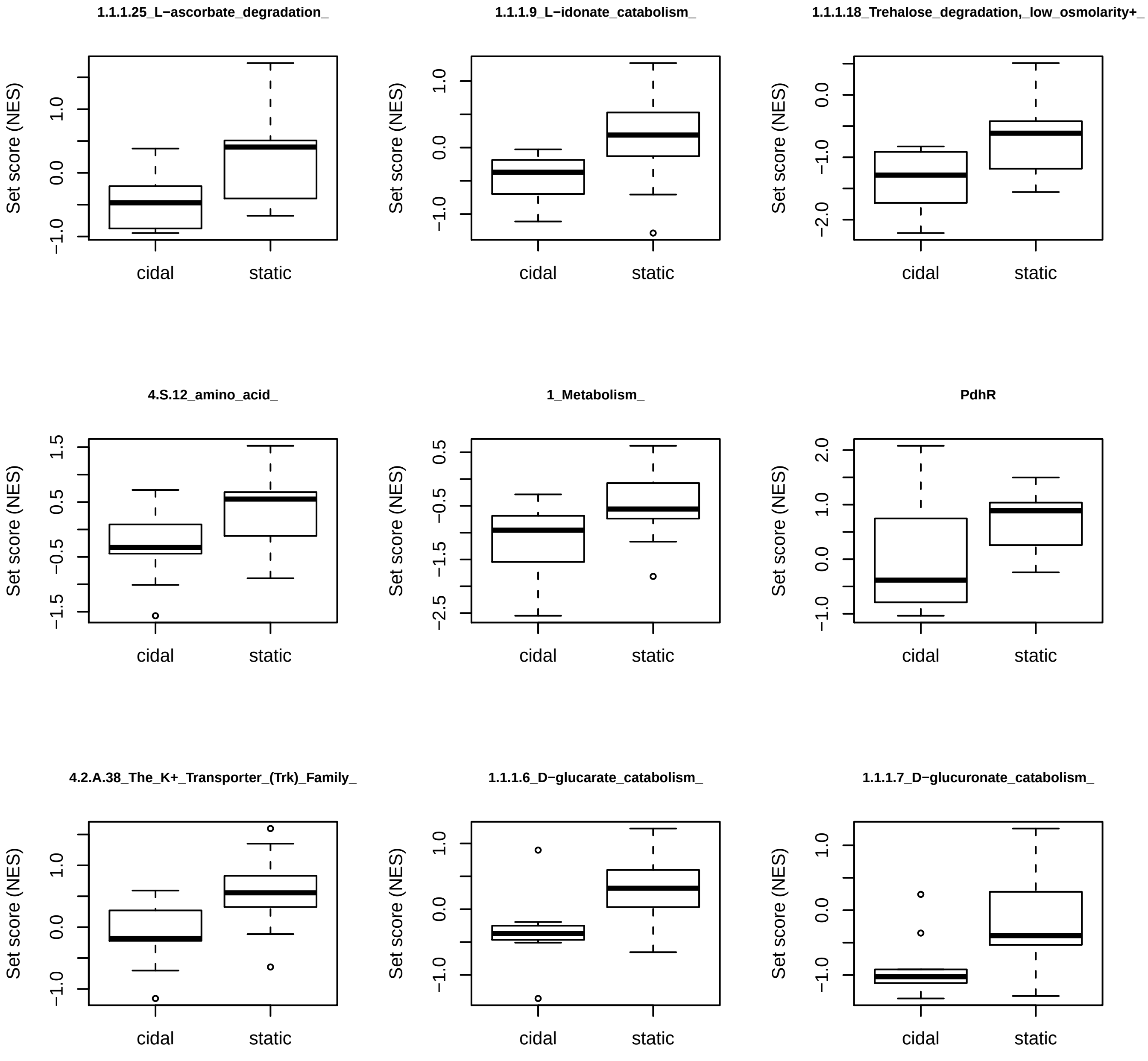
1.1.1.25_L−ascorbate_degradation_
1.1.1.9_L−idonate_catabolism_
1.1.1.18_Trehalose_degradation,_low_osmolarity+_
4.S.12_amino_acid_
1_Metabolism_
PdhR
4.2.A.38_The_K+_Transporter_(Trk)_Family_
1.1.1.6_D−glucarate_catabolism_
1.1.1.7_D−glucuronate_catabolism_
Set score (NES)
cidal
static

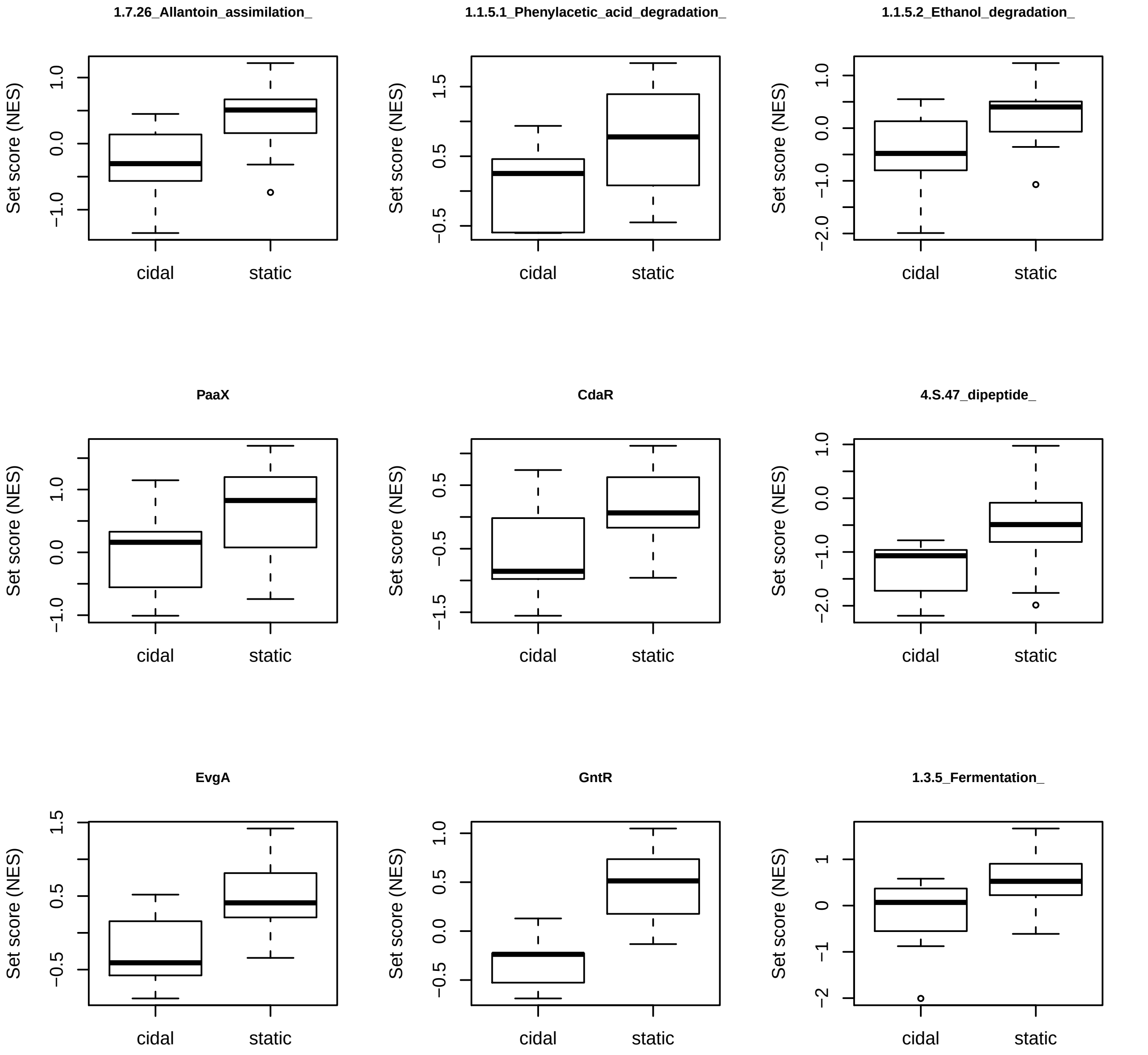

1.7.26_Allantoin_assimilation_
Set score (NES)
−1.0
0.0
1.0
cidal
static
1.1.5.1_Phenylacetic_acid_degradation_
Set score (NES)
−0.5
0.5
1.5
cidal
static
1.1.5.2_Ethanol_degradation_
Set score (NES)
−2.0
−1.0
0.0
1.0
cidal
static
PaaX
Set score (NES)
−1.0
0.0
1.0
cidal
static
CdaR
Set score (NES)
−1.5
−0.5
0.5
cidal
static
4.S.47_dipeptide_
Set score (NES)
−2.0
−1.0
0.0
1.0
cidal
static
EvgA
Set score (NES)
−0.5
0.5
1.5
cidal
static
GntR
Set score (NES)
−0.5
0.0
0.5
1.0
cidal
static
1.3.5_Fermentation_
Set score (NES)
−2
−1
0
1
cidal
static

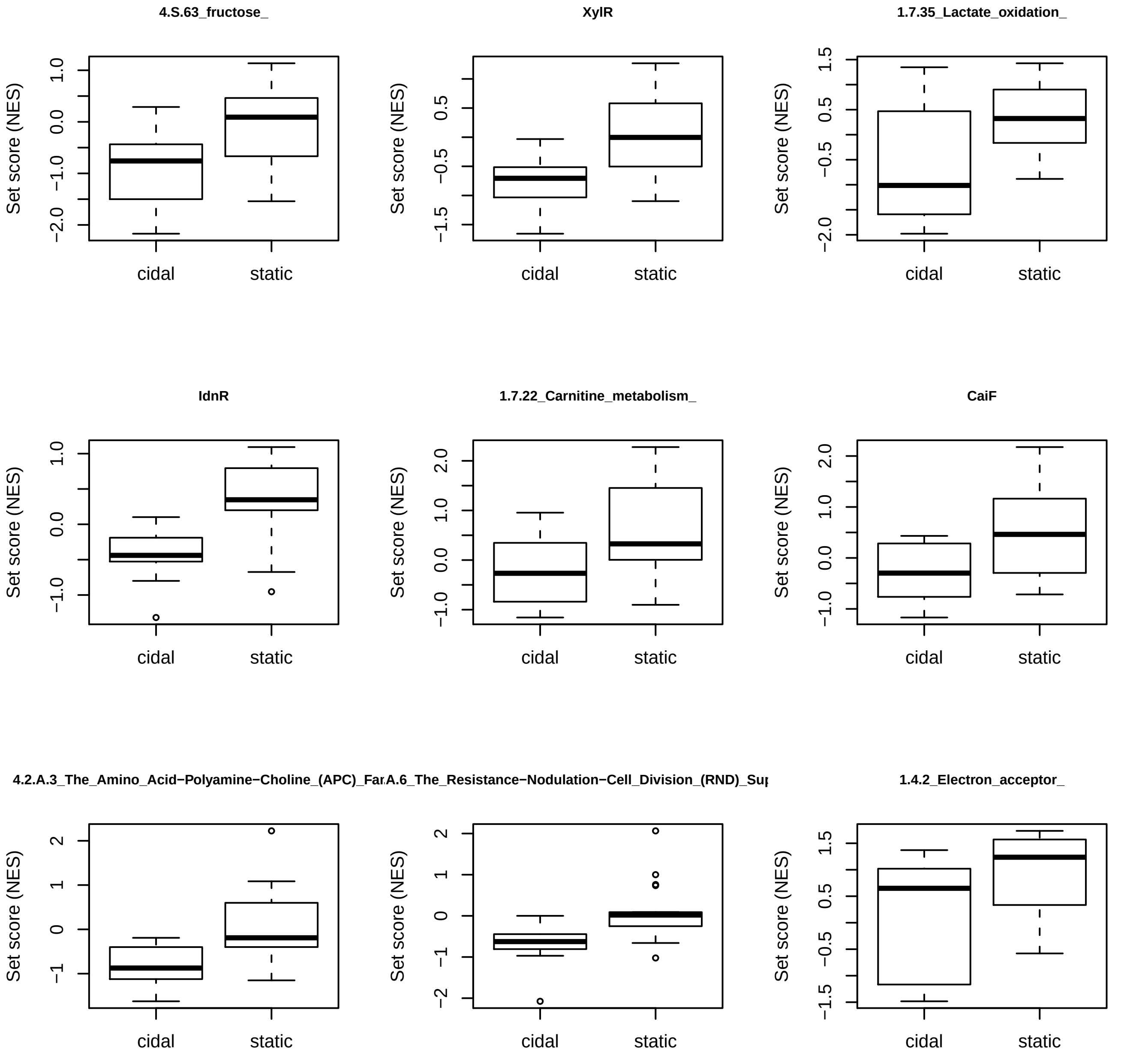
4.S.63_fructose_
XylR
1.7.35_Lactate_oxidation_
IdnR
1.7.22_Carnitine_metabolism_
CaiF
4.2.A.3_The_Amino_Acid−Polyamine−Choline_(APC)_Fa
A.6_The_Resistance−Nodulation−Cell_Division_(RND)_Su
1.4.2_Electron_acceptor_
Set score (NES)
cidal
static

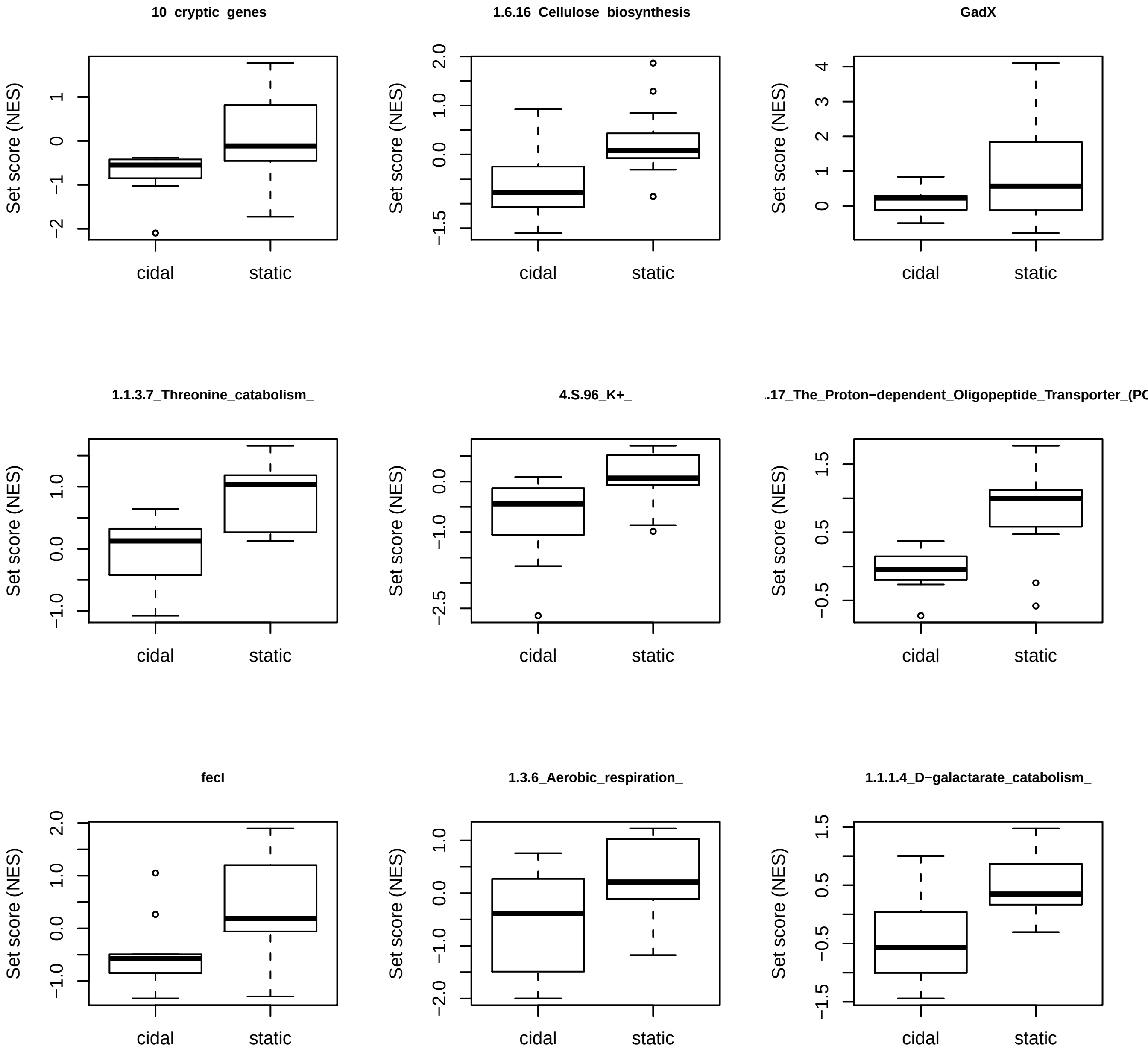

10_cryptic_genes_
1.6.16_Cellulose_biosynthesis_
GadX
1.1.3.7_Threonine_catabolism_
4.S.96_K+_
.17_The_Proton−dependent_Oligopeptide_Transporter_(PO
fecI
1.3.6_Aerobic_respiration_
1.1.1.4_D−galactarate_catabolism_
Set score (NES)
cidal
static

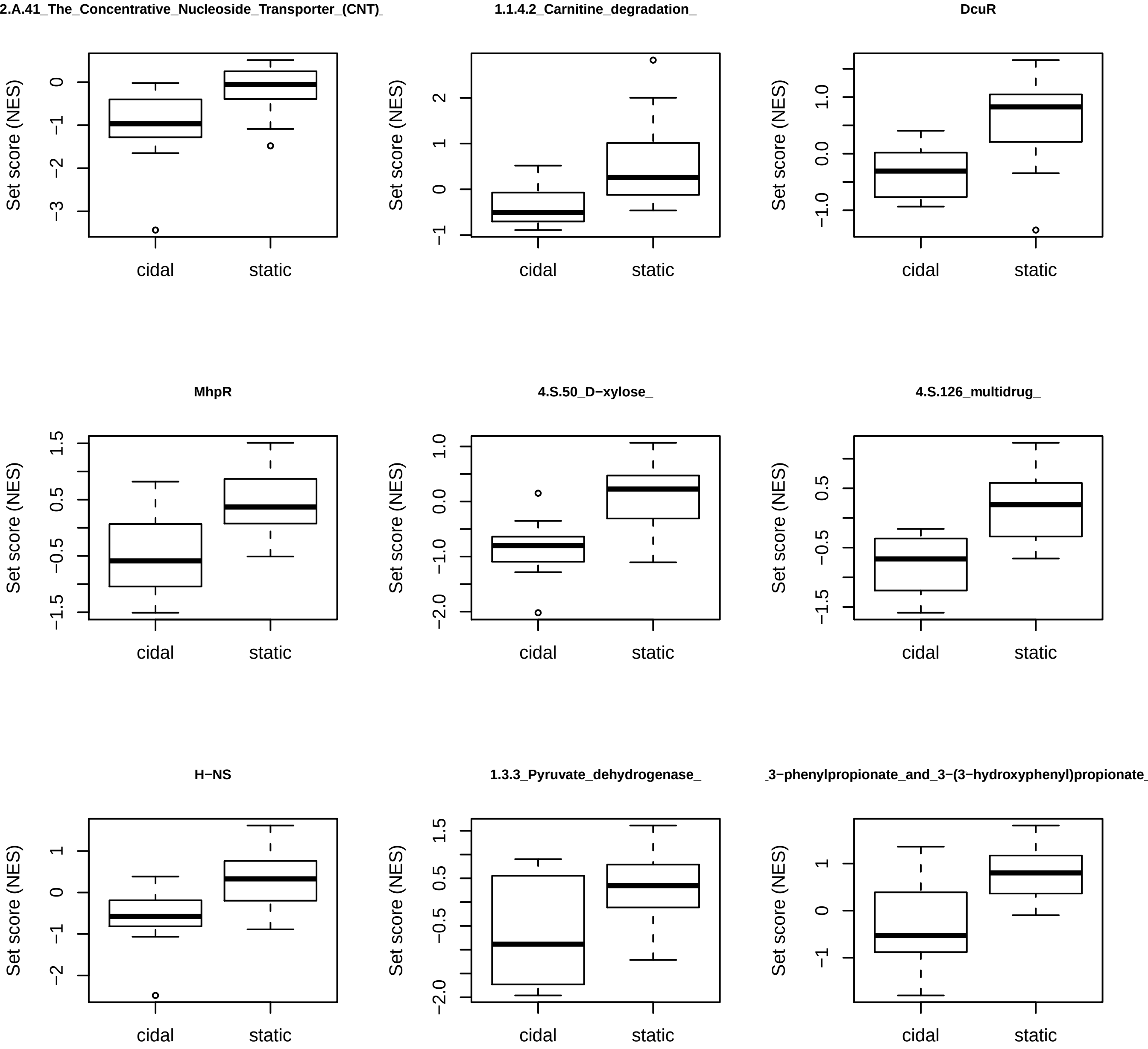
2.A.41_The_Concentrative_Nucleoside_Transporter_(CNT)_
1.1.4.2_Carnitine_degradation_
DcuR
MhpR
4.S.50_D−xylose_
4.S.126_multidrug_
H−NS
1.3.3_Pyruvate_dehydrogenase_
3−phenylpropionate_and_3−(3−hydroxyphenyl)propionate_
Set score (NES)
cidal
static

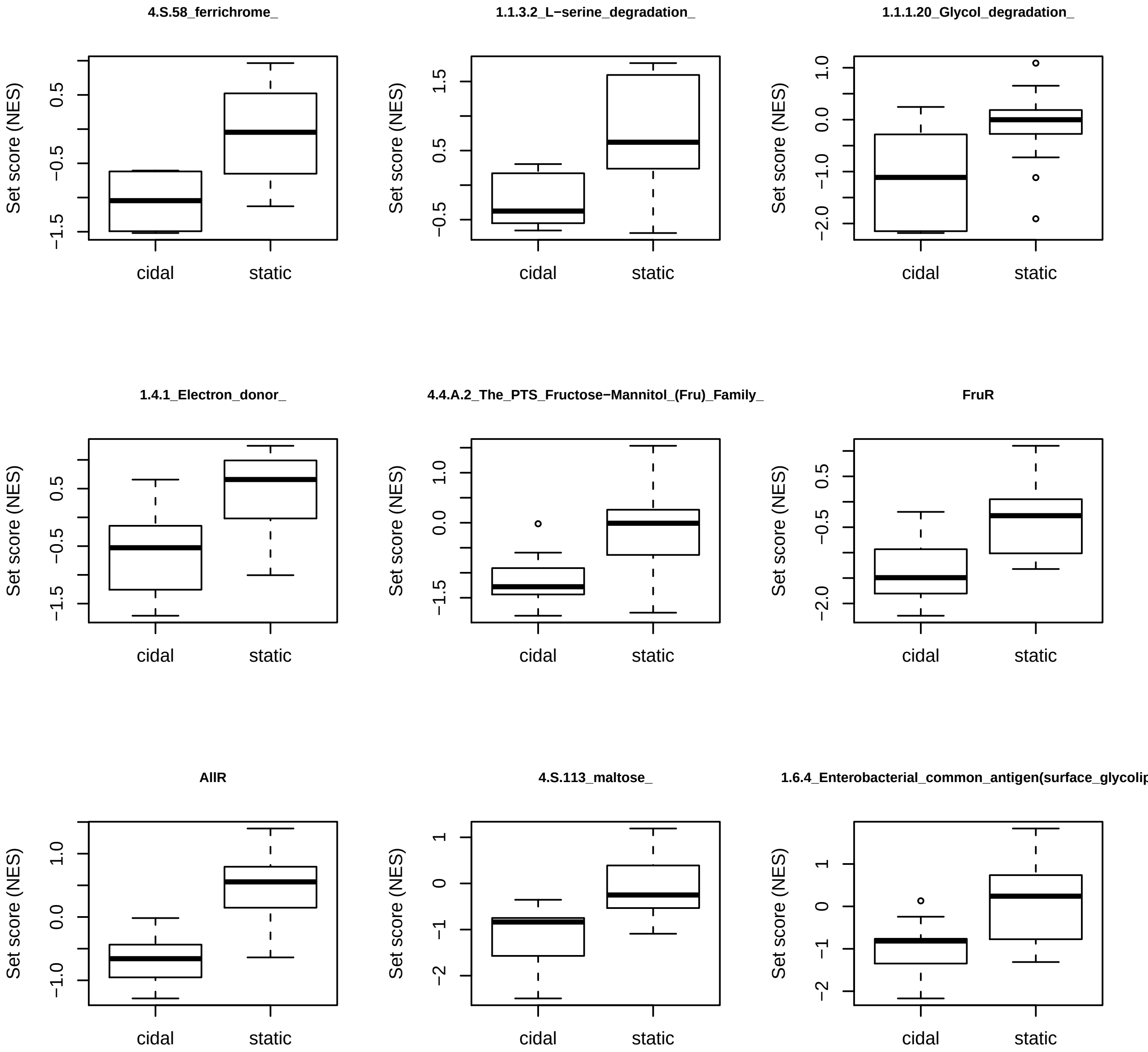
4.S.58_ferrichrome_
1.1.3.2_L−serine_degradation_
1.1.1.20_Glycol_degradation_
1.4.1_Electron_donor_
4.4.A.2_The_PTS_Fructose−Mannitol_(Fru)_Family_
FruR
AllR
4.S.113_maltose_
1.6.4_Enterobacterial_common_antigen(surface_glycolip
Set score (NES)
cidal
static

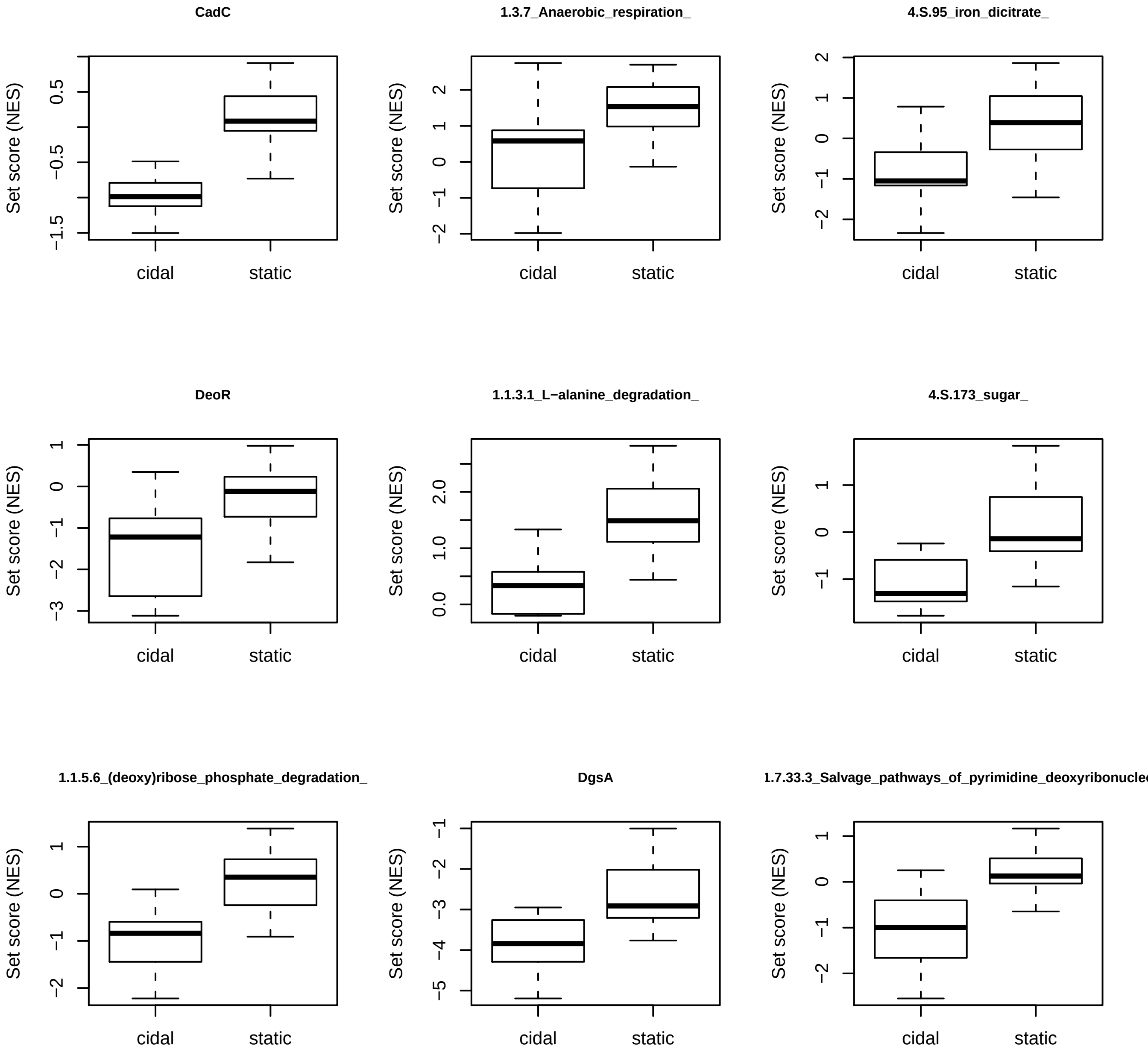
CadC
Set score (NES)
−1.5
−0.5
0.5
cidal
static
1.3.7_Anaerobic_respiration_
Set score (NES)
−2
−1
0
1
2
cidal
static
4.S.95_iron_dicitrate_
Set score (NES)
−2
−1
0
1
2
cidal
static
DeoR
Set score (NES)
−3
−2
−1
0
1
cidal
static
1.1.3.1_L−alanine_degradation_
Set score (NES)
0.0
1.0
2.0
cidal
static
4.S.173_sugar_
Set score (NES)
−1
0
1
cidal
static
1.1.5.6_(deoxy)ribose_phosphate_degradation_
Set score (NES)
−2
−1
0
1
cidal
static
DgsA
Set score (NES)
−5
−4
−3
−2
−1
cidal
static
1.7.33.3_Salvage_pathways_of_pyrimidine_deoxyribonucle
Set score (NES)
−2
−1
0
1
cidal
static

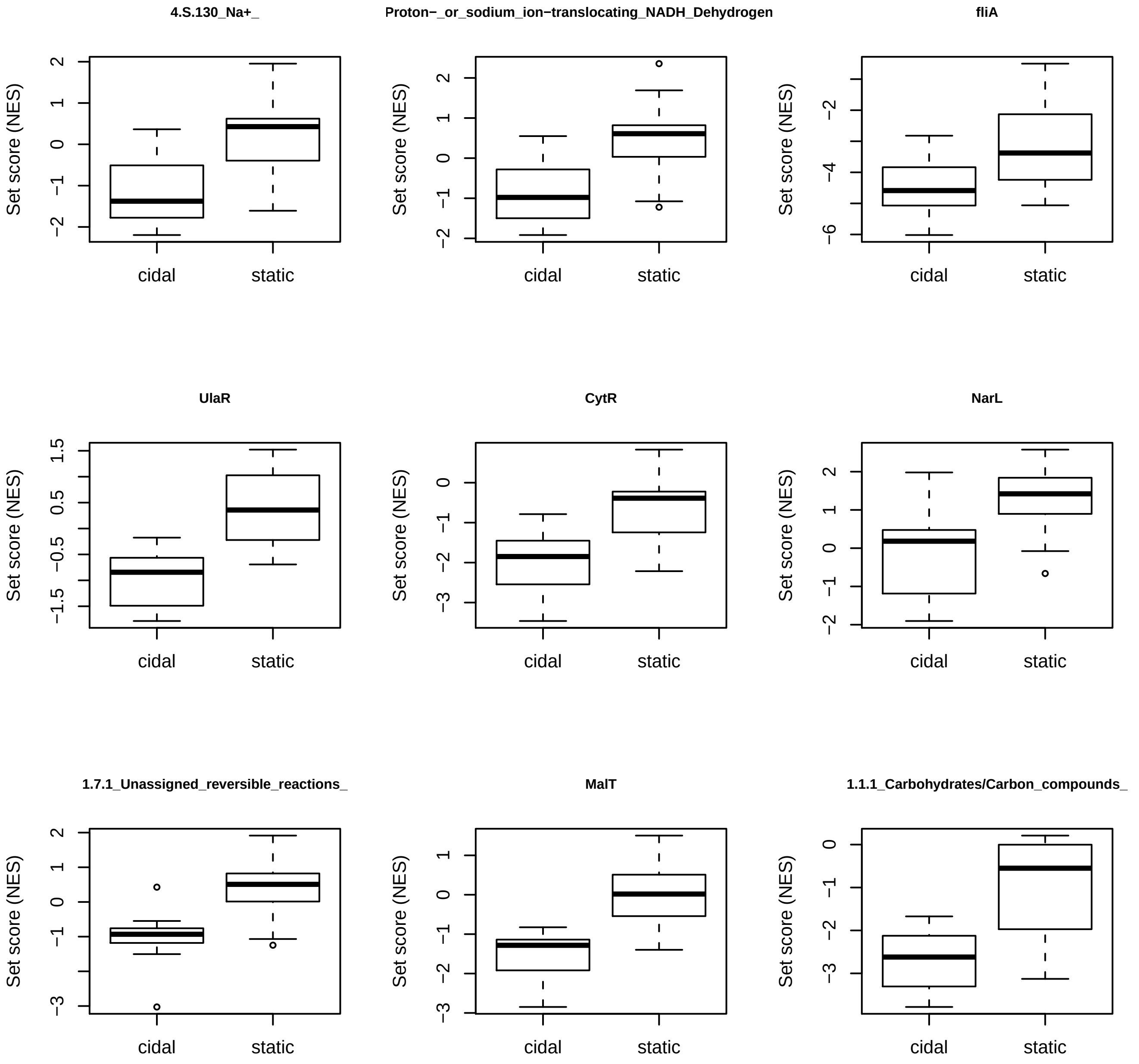
4.S.130_Na+_
Proton−_or_sodium_ion−translocating_NADH_Dehydrogen
fliA
UlaR
CytR
NarL
1.7.1_Unassigned_reversible_reactions_
MalT
1.1.1_Carbohydrates/Carbon_compounds_
Set score (NES)
cidal
static
