# Supplementary figures and images for: The association of DNA damage response and nucleotide level modulation with the antibacterial mechanism of the anti-folate drug Trimethoprim
Source: BMC Genomics. 2011 Nov 28;12:583. doi: 10.1186/1471-2164-12-583 (PMC3258297; doi:10.1186/1471-2164-12-583)

Comparison of regression based and AUC test

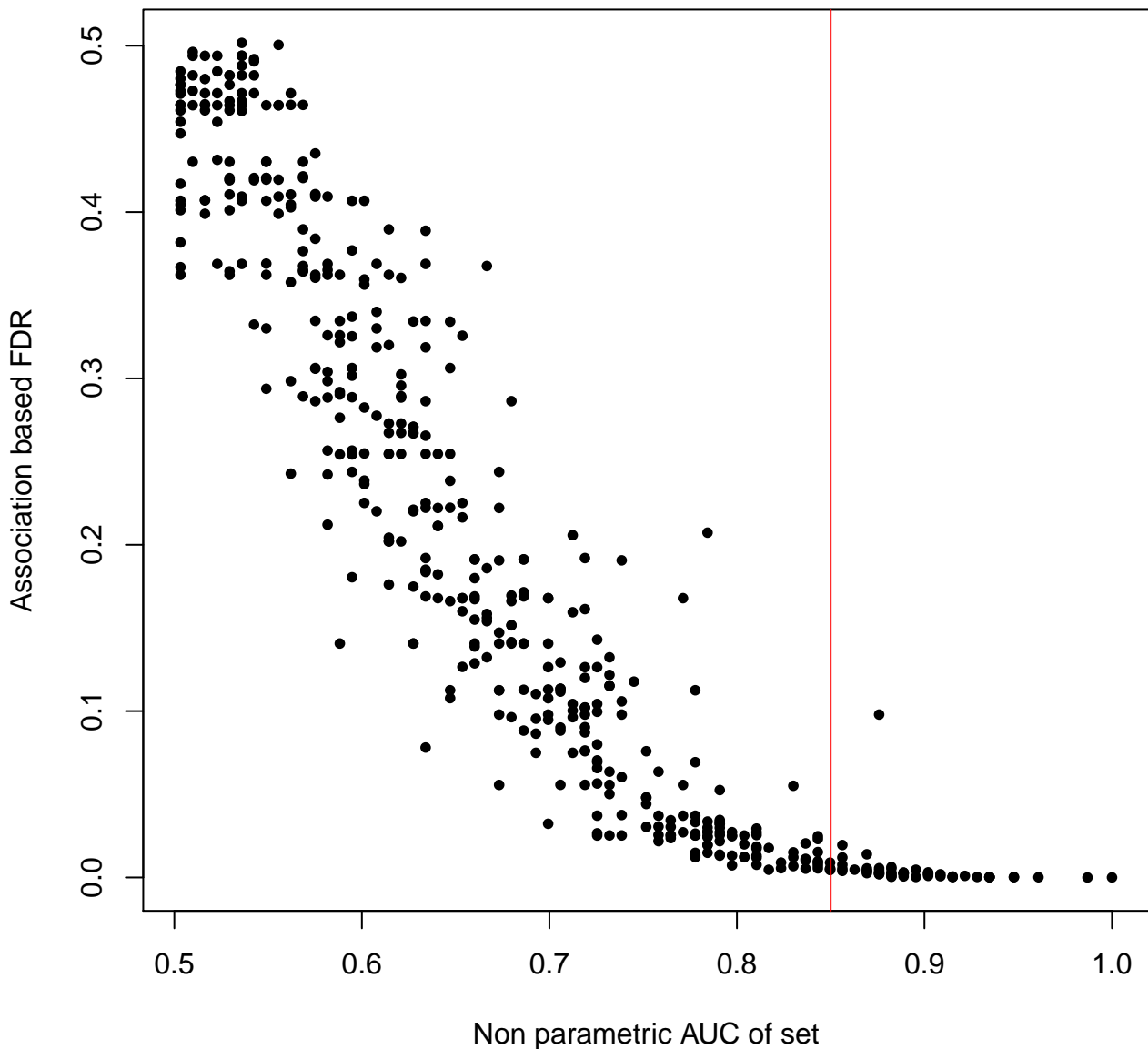

Supplement: Additional file 6 — Comparison of non-parametric (AUC) and parametric (linear model FDR) significant tests for sets. Scatter plot of AUC scores and False Discovery Rates (FDR) from the linear model indicating strong correlation between the two measures of association between set scores and cell phenotype. [file 1471-2164-12-583-S6.PDF]
