# Supplementary material for: The association of DNA damage response and nucleotide level modulation with the antibacterial mechanism of the anti-folate drug Trimethoprim
Source: BMC Genomics. 2011 Nov 28;12:583. doi: 10.1186/1471-2164-12-583 (PMC3258297; doi:10.1186/1471-2164-12-583)
Supplement: Additional file 7 — O.D. values of deo mutants after TMP treatment in M9 and LB media. A) LBTMP treatment. Increase in O.D. (600 nm) after 50 μg/ml TMP treatment of wild-type MG1655 strain (blue), deoA (red), deoC (green) and deoR (purple) knock-out mutants in LB medium. TMP was added to respective cultures grown from overnight inoculum to mid-log phase in LB medium at O.D. 0.37-0.42 (Time 0 hr). B) M9TMPAdAA treatment. Increase in O.D. (600 nm) after 25 μg/ml TMP treatment of wild-type MG1655 strain (blue), deoA (red), deoC (green) and deoR (purple) knock-out mutants in M9 medium. TMP was added to respective cultures grown from overnight inoculum to mid-log phase in M9 medium at O.D. 0.25-0.35 (Time 0 hr). [file 1471-2164-12-583-S7.PDF]

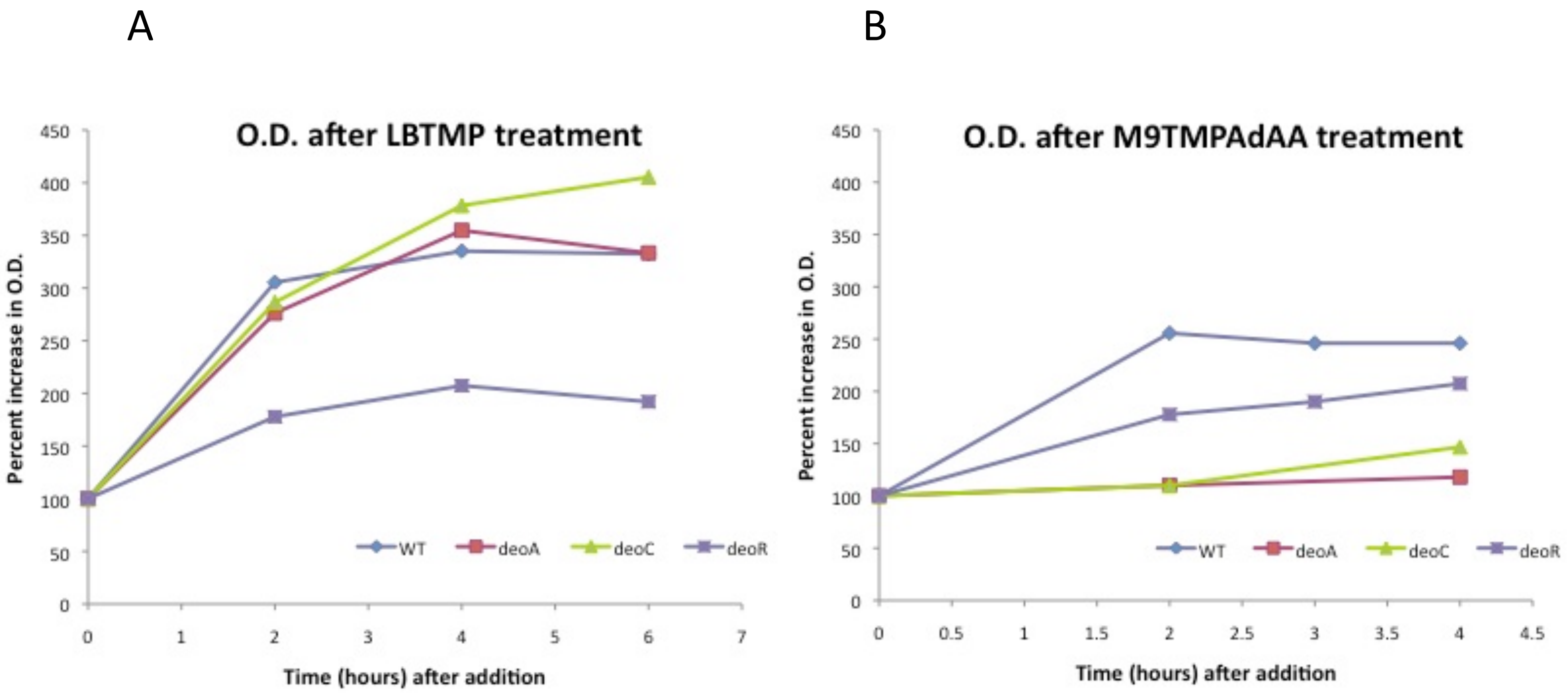

Supplementary File 6: Effect of TMP treatment on O.D. of *deo* strains.

A)LBTMP treatment. Increase in O.D. (600 nm) after 50 µg/ml TMP treatment of wild-type MG1655 strain (blue), *deoA* (red), *deoC* (green) and *deoR* (purple) knock-out mutants in LB medium. TMP was added to respective cultures grown from overnight inoculum to mid-log phase in LB medium at O.D. 0.37-0.42 (Time 0 hr).

B) M9TMPAdAA treatment. Increase in O.D. (600 nm) after 25 µg/ml TMP treatment of wild-type MG1655 strain (blue), *deoA* (red), *deoC* (green) and *deoR* (purple) knock-out mutants in M9 medium. TMP was added to respective cultures grown from overnight inoculum to mid-log phase in M9medium at O.D. 0.25-0.35 (Time 0 hr).
